# Traditional and Non-Conventional Methods of Pre-Treatment of Biological Raw Materials for Drying Food

**DOI:** 10.3390/foods15173106

**Published:** 2026-09-01

**Authors:** Dorota Nowak, Ewa Jakubczyk

**Affiliations:** Department of Food Engineering, Institute of Food Sciences, Warsaw University of Life Sciences, 02-776 Warsaw, Poland; dorota_nowak@sggw.edu.pl

**Keywords:** mechanical, physicochemical, thermal, and non-thermal methods, quality properties, drying efficiency, fruits, vegetables, blanching, ohmic heating, enzymes

## Abstract

Pre-treatment before drying is a crucial phase in food processing. This review highlights key traditional and innovative pre-treatment methods, focusing on their mechanisms of action at the cellular and tissue levels. This discourse examines innovative technologies with significant promise across various applications, as well as widely recognised methodologies. These include cold plasma, ultrasonication, pulsed electric fields, high-pressure processing, UV-C light, pulsed light, and coating techniques. It addresses biological components that act as barriers to mass transfer, thereby significantly influencing the efficiency of moisture evaporation. Understanding these interactions is crucial for optimising drying processes and enhancing the quality of dried food. Key pre-treatment parameters that affect outcomes are analysed and must be tailored to the specific characteristics of biological materials, which often require individualised adjustments. Each method is evaluated against goals like accelerated drying, microbiological purity, enzyme inactivation, and preservation of active components, emphasising the need for a targeted optimisation approach. The classification of biological materials into distinct categories has been proposed based on their structural and integumentary characteristics. The research outlines effective methodologies for each material type and pre-treatment purpose. This analysis also incorporates an economic perspective, considering the initial investment and operational costs of implementing the proposed methods.

## 1. Introduction

To achieve an economically viable drying process with high-quality products, it is essential to understand the parameters influencing the drying outcome. This knowledge is crucial for optimising the process and achieving desired product characteristics [1]. From a process engineering perspective, the design of the evaporation process aims to achieve high drying rates. High drying rates can be achieved by a high driving force, which is the difference in water vapour pressure between the surface of the dried material and its surroundings, or by minimising the resistance to water movement. A significant driving force for these elevated drying rates is the difference in water vapour pressure between the surface of the dried material and its surrounding environment. Additionally, minimising the resistance to moisture movement also enhances drying efficiency. The total resistance to water transport consists of two main components: the resistance to water diffusion within the dried material and the resistance to the penetration of water vapour into the surrounding environment [2]. Notably, this latter component significantly influences the drying process, especially during the initial stages of evaporation. Your insights on this topic are greatly appreciated [3]. It can be modified using parameters related to dryer operation (e.g., airflow rate, humidity, temperature, and pressure reduction), thereby increasing the mass transfer coefficient to the surroundings [3,4]. During the second drying period, the dominant resistance is the resistance to water diffusion within the material as it moves towards the evaporation surface. This resistance arises from both the transport of liquid water driven by the concentration difference induced by evaporation and the resistance to water vapour transport within the material structure [2]. Therefore, the movement of water in a material is influenced by its chemical composition, material structure (porosity, pore size and its distribution, the way water binds to the material, and the share of bound water in relation to free water, the characteristic shape and dimensions determining the mass transport path, the presence of cell membranes and skin (especially in berries) [5]. For plant and animal raw materials, the presence of cell membranes is a very important factor influencing resistance to mass transport. Their biological role is to regulate the cell’s water content and prevent excessive water loss [2]. Hence, the presence of an integral cell membrane limits mass transport. To effectively prepare material for drying, careful regulation of its shape and dimensions is necessary. This process involves disrupting cellular membranes and creating a suitable structural framework to optimise drying [1].

Numerous qualitative changes occur during pre-treatment and drying. Cutting or damaging the material releases enzymes and triggers defence responses, which can cause darkening or degradation of components, leading to undesirable changes in quality. During drying, the material is exposed to high temperatures, increased water activity, and often significant contact with atmospheric oxygen. These conditions can lead to further deterioration in quality, including microbial growth. Furthermore, a significant proportion of flavour and aroma compounds are volatile, which can lead to their degradation or loss during the drying process, especially when conducted under reduced pressure.

To achieve economically efficient drying processes while minimising undesirable alterations, it is imperative to possess a comprehensive understanding of the material properties involved. Anticipating changes during drying is essential for preserving material quality and integrity.

Employing both traditional and modern methods can help reduce negative quality changes. Methods of pre-treatment of plant and animal raw materials with a pre-cellular structure are used to:

- Shorten drying time: achieved mainly by cutting, grinding, or increasing the evaporative surface area to reduce the transport path and mass and heat transfer resistance, or by disrupting cell membranes to lower water diffusion resistance [1,5,6];
- Improve visual quality: deactivate enzymes (e.g., polyphenol oxidase) to limit browning, shrinkage, and other undesirable changes [7];
- Enhance sensory quality [8];
- Maintain or enhance nutritional value by adding functional ingredients [9];
- Improve functional properties, such as increased rehydration capacity and enhanced chemical composition [10];
- Reduce energy consumption: shorter drying times lower overall energy requirements [11];
- Increase food safety: certain methods reduce microbiological load or chemical residues [12].

When assessing pre-treatment methodologies for drying, it is crucial to categorise materials into two distinct classifications: liquids and solids. Solid materials typically include plant or animal tissues and their products, which may still contain cells with intact membranes. The present study focuses on preparation techniques that account for the biological characteristics inherent in these raw materials.

The characteristics of raw materials, which arise from their biological nature, play a crucial role in various processes. Key factors include chemical composition, the presence of skin, cell membranes, air spaces, and overall structure. These features significantly influence the transport of water. Additionally, both plant and animal raw materials can vary even within the same variety due to factors such as maturity, agricultural practices, and treatment methods. Therefore, when selecting an effective pre-treatment method and its parameters, it is essential to tailor them to the specific material rather than merely copying the parameters recommended in the literature.

**Figure 1:**
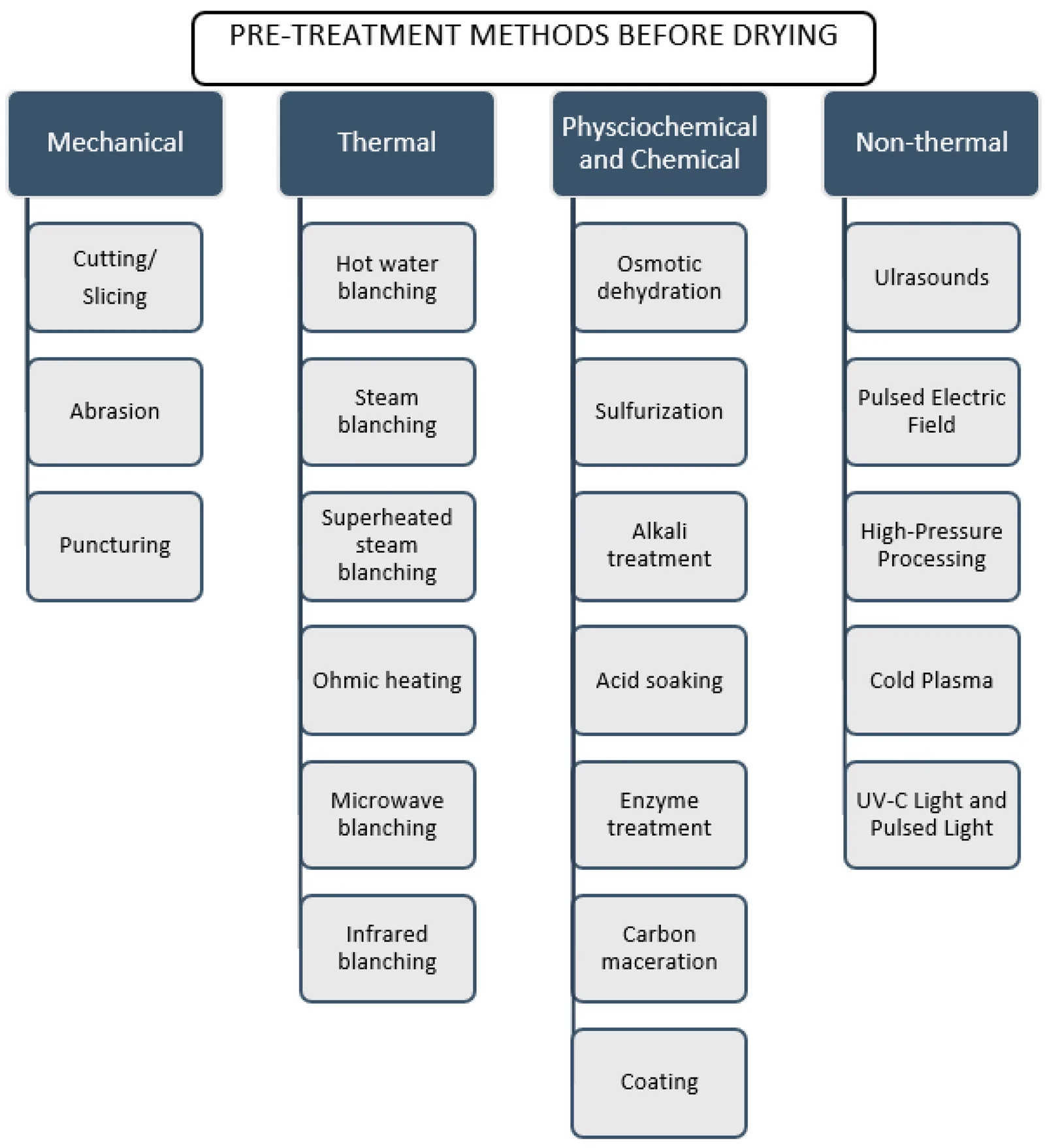
Classification of pre-treatment methods before drying.

This review presents a detailed analysis of both traditional and modern (unconventional) methods for food pre-treatment before drying. The study aimed to identify key factors influencing the selection of appropriate processing methods for a variety of solid products. Both traditional and novel approaches to processing can be classified into several categories, including mechanical, physicochemical, chemical, thermal, and non-thermal methods (Figure 1). It is important to note that this classification is not strictly defined; for example, enzymatic techniques and various coating processes may also be regarded as non-thermal methods.

It summarises the current state of knowledge on the mechanisms underlying pre-treatment and its impact on critical quality attributes of dried food, including the retention of nutrients and bioactive compounds, improvements in colour and texture, rehydration properties, and other physicochemical parameters. Furthermore, strategies for reducing drying time and energy consumption through pre-treatment were analysed. In the present study, we undertook a comprehensive systematic review utilising databases such as Scopus and Web of Science, covering literature from 1998 to 2026. The search employed a range of keywords including ‘pre-treatment’, ‘drying’, ‘chemical methods’, ‘psychochemical methods’, ‘thermal methods’, ‘mechanical methods’, as well as specific techniques like cold plasma, pulsed electric fields, osmotic dehydration, ultrasounds, high pressure, enzymatic pre-treatment, ohmic heating, radiation, UV-C light, pulsed light, and carbon dioxide treatment. Additional terms such as ‘drying time’, ‘drying kinetics’, ‘quality’, ‘bioactive compounds’, ‘colour’, and ‘texture’ were also incorporated using Boolean operators to refine the search. The initial screening process involved evaluation of articles based on titles and abstracts, followed by a thorough full-text review according to predefined inclusion and exclusion criteria. Research articles, review papers, and book chapters were considered in this analysis. Notably, we excluded studies that did not provide sufficient information on the technical parameters of pre-treatment or drying processes. Furthermore, manual searches of reference lists were conducted to identify additional relevant literature.

We aimed not only to describe specific processing methods but also to highlight factors that may limit their application before drying. A thorough understanding of these methods and their influence on both the drying process and the final product can be essential for selecting the appropriate processing technique for specific raw materials. The study also highlighted less common but promising processing methods that have not been widely discussed in previous literature on this topic.

## 2. Traditional Pre-Treatment Before Drying

The oldest and most common methods of preparing material for drying include grinding (slicing), blanching, soaking in protective solutions, or freezing, which is necessary before freeze-drying. These methods primarily apply to fruits and vegetables.

Slicing (giving a specific geometric shape) or calibrating small fruits standardises the size, thickness, shape, or particle distribution of the components. This plays a fundamental role in preparing fruits and vegetables for drying [13,14]. Its purpose is to shorten and smoothen the paths for mass and heat transfer, as well as to achieve uniformity. This ensures shorter drying times and uniform drying of the entire batch. Additionally, material in the form of chips or other particles can create a porous layer through which drying air or water vapour can flow. Cutting can also eliminate or reduce the barrier created by the skin [5]. For raw materials with an anisotropic structure (e.g., banana, carrot, parsley, cucumber), the cutting direction is also important [10]. However, excessive grinding can cause leakage of cellular fluid, leading to the loss of valuable nutrients.

Thermal blanching involves short-term treatment of the material with hot water, air, or steam. This should inactivate enzymes such as polyphenoloxidase (PPO) and peroxidase (POD), which cause enzymatic browning or oxidation, and thus quality deterioration during drying and storage [15]. Other goals of blanching include reducing microbial load, softening the tissue, and partially denaturing cell membranes [16]. Blanching also removes air from intercellular spaces, thereby reducing the resistance to heat diffusion within the dried material. Steam blanching improved the quality of dried apple slices, particularly by increasing phytochemical concentration and antioxidant activity compared with fresh [17]. It may also affect the nanostructure of pectin [18]. Blanching may increase the availability of phenolic compounds. However, it may also leach water-soluble nutrients such as vitamins B1 and B2, tocopherols [19,20] and pigments. It may also lead to excessive softening, especially in fruits [21].

Soaking in protective solutions is a traditional method for preparing materials for drying. Soaking in citric acid solutions and sulfurization (SO_2_/sulphites) are the two most commonly used chemical treatments before drying fruits and vegetables. Both are intended to inhibit browning reactions, improve colour, increase drying efficiency, and improve the quality of the final product. Still, they operate differently and have distinct technological and sensory effects [3]. While citric acid solutions produce a light colour close to natural colour, sulfurization produces an intense, often unnaturally light colour. SO_2_ is frequently used in food processing for its technological properties. It acts as an antioxidant, bleaching agent, colour stabiliser, inhibitor of enzymatic discolouration and non-enzymatic browning, and antimicrobial preservative [22]. It is widely used for fruits and vegetables, fruit juices and concentrates, syrups, wines, and jams, and, to a lesser extent, for seafood, meat products, sausages, and mushrooms [23]. SO_2_ treatment has been shown to effectively eradicate moulds, yeasts, and pathogens on the surfaces of various fresh fruits and vegetables [24]. This method has demonstrated particular efficacy in controlling microbial populations on grapes [25], lychees [26], citrus fruits, and dried apricots [27,28].

The use of sulphur dioxide (SO_2_) in the food industry serves two main purposes: it significantly extends the shelf life of various products. It enhances their safety for consumption by reducing microbial load. SO_2_ has broad-spectrum antimicrobial properties, effectively inhibiting a wide range of foodborne and post-harvest pathogens. This characteristic not only improves the safety of food products but also plays a crucial role in maintaining their quality during storage and distribution. Despite its benefits, the use of SO_2_ is regulated due to potential residues in treated foods. The main concern is the presence of chemical residues and their safety implications. Many countries have established maximum permitted levels to keep consumer exposure within safe thresholds. Excessive consumption of SO_2_ may adversely affect health, particularly for sensitive individuals, who might experience reactions in the respiratory, cardiovascular, nervous, and gastrointestinal systems. Additionally, while free SO_2_ has been well studied, the toxicological significance of many bound forms of SO_2_ remains poorly understood, raising concerns about their long-term safety [23].

Freezing is a necessary preliminary operation before freeze-drying. Its purpose is to freeze out all free water. The freezing speed is also important for the quality of the finished lyophilizate [29]. Slow freezing results in the formation of large ice crystals that destroy cellular structure. This effect can be enhanced by freezing and thawing. This creates favourable conditions for recrystallisation, producing larger crystals and leaving larger pores after sublimation [29]. This can facilitate the removal of water from compactly structured tissues. Rapid freezing is more beneficial for materials with a relatively loose, porous cellular structure. Small crystals sublimate more quickly and do not disrupt the cellular structure.

Conventional methods often require balancing process efficiency with nutrient retention and sensory qualities. Traditional approaches may not fully address the functional and sensory attributes of the final dried product.

### 2.1. Mechanical Treatment

The implementation of physical pre-treatment processes for plant materials before the drying phase has been shown to reduce both drying time and energy consumption effectively. Moreover, this approach helps preserve the overall quality of the end products [30,31]. Processes involving cutting, grading and abrasion of fruits and vegetables can be categorised among physical methods. Mechanical pre-treatment can also involve making pinholes or drill holes in whole or sliced fruits. Needle micro-penetration is a process that creates pores in the epidermis of fruit. The pitting effect reduces skin resistance, damages its integrity, partially destroys cell membranes, and creates pores that facilitate water migration to the surface, thereby reducing both drying time and associated costs. This method offers a viable alternative to traditional chemical drying techniques, with potential benefits for efficiency and product quality [32,33]. During drying, foods shrink, and holes also reduce their size and become less noticeable in the dried material [34]. This pre-treatment is recommended to induce partial skin damage, thereby enhancing the efficiency of water removal. Yong et al. [34] observed that mechanical pre-treatment of potato, cassava, dragon fruit, and red chilli before drying increased the drying rate, and this effect was further accelerated by increasing the diameter and density of the holes. The overall changes in colour and the reduction in volume during the drying process of samples with holes and those without were comparable. Enhancements in the structure of grape berries, such as perforations, resulted in a more than twofold rise in the effective diffusion coefficient during the convective drying process at 50 °C [35]. The needle micro-penetration method was also used before a conventional drying of grapes. The findings of this investigation indicated that the applied pre-treatment significantly improved colour retention and facilitated enhanced mass transfer during the drying process. This effect was particularly pronounced even at lower temperatures compared with fruits dipped in an alkaline solution [33]. Needle penetration has been proposed as an effective pre-treatment method to enhance blueberry freeze-drying, resulting in a 2.5-fold reduction in drying time. Furthermore, the biochemical analysis revealed that the needle-treated blueberries retained anthocyanin and polyphenol concentrations comparable to those in untreated dried fruits [36].

Drying many berries, plums, and cherries is challenging because of the waxy coating on their surfaces. This layer is a major barrier to moisture removal during drying. One technique for removing or disrupting a wax layer is abrasion. The abraded samples were mainly obtained using a motorised rotating drum with an inner surface covered with sandpaper or other abrasive coating [37,38]. Di Matteo et al. [39] reported that applying abrasion to the grapes decreased drying time from 90 to 35 h. However, the observed colour of abraded berries was found to be darker in comparison to that of chemically treated berries. This phenomenon can be attributed to increased enzymatic browning in the abrasive-treated samples [39]. Similarly, dried red grapes obtained using the abrasive pre-treatment had a 33% shorter drying time than untreated grapes. Furthermore, samples exhibiting abraded peels demonstrated reduced shrinkage and superior rehydration capacity compared to their untreated dried fruit counterparts [40]. The implementation of an abrasive pre-treatment before drying goji berries at 50 °C significantly reduced drying time, from 31.2 h to 17.4 h. This indicates that the pre-treatment may enhance moisture removal efficiency during drying. Consequently, this method facilitated the enhanced preservation of colour and bioactive constituents, including carotenoids [38] and phenolic compounds such as caffeic and chlorogenic acids [37], compared with untreated dried fruit samples. The reduced exposure to heat and oxygen in the abraded fruits resulted in greater retention of colour and bioactive compounds than in those subjected to conventional drying without this pre-treatment.

Mechanical pre-treatment, which can increase the active surface area for mass transfer, involves cutting samples into different shapes, such as halves, quarters, cylinders, and cubes [41]. Persimmons were prepared in various geometrical forms, specifically cylinders, slices, and sectors. The findings indicated that the effective diffusivity of water was significantly greater during the drying of slices than during the drying of sectors. Interestingly, alterations in size and shape did not substantially affect the colour or textural characteristics of the dried products. The choice of shape should be determined by the intended application of the dried snacks rather than the physical properties measured [42]. Grabowski et al. [43] observed that halving the cranberries significantly increased water diffusion from the cut surfaces, roughly 100 times that of diffusion through the skin. Smaller, thinner pieces can typically dry more quickly and may have less uneven shrinkage, resulting in a better texture and appearance of the final product [44]. Cutting fruit before drying has significant implications for the texture of the dried product, primarily by altering the structural integrity of cellular tissue. The degree of shrinkage observed in samples with varying geometries can differ markedly, thereby influencing their textural characteristics, including hardness and chewiness [41,42].

The pre-treatment of raw materials, particularly through methods such as cutting or grinding, presents significant challenges, including fluid leakage and the concomitant loss of minerals, vitamins, and bioactive compounds. An increase in surface area enhances oxygen accessibility, thereby potentially facilitating oxidative degradation processes. Furthermore, there exists a heightened risk of microbial contamination associated with these techniques. In certain instances, cutting particular raw materials may be inadvisable; for example, maintaining the integrity of products such as berries is often preferable to preserve their nutritional value.

Nonetheless, both peel abrasion and skin puncturing are impractical at an industrial scale due to the cumbersome, labour-intensive, and costly processes associated with these pre-treatments [30]. During the peeling process, mechanical damage can arise from factors such as abrasion and puncturing. In industrial environments where sophisticated machinery operates at high speeds, numerous adverse phenomena may manifest. The products are subjected to various unwanted mechanical stresses, including compression, impact, shearing, and vibration [45]. These stresses can lead to bruising and cracking of the biological material. A compromise in tissue integrity can result in leakage, which significantly increases the risk of microbial spoilage, mould and bacterial contamination during large-scale handling on industrial production lines. The implementation of these mechanical methods within a production-line context presents significant challenges. Therefore, it may be more feasible to explore tissue perforation through alternative techniques such as pulsed electric fields (PEFs), laser applications, or enzymatic treatments. These methods may provide a more efficient and effective way to achieve the desired outcomes in tissue modification.

In our assessment, methodologies such as abrasion and skin puncturing may be effective for small enterprises with lower throughput. In such contexts, the application of slower-speed machinery could be advantageous, as it would mitigate tissue damage while maintaining operational efficiency.

### 2.2. Thermal Treatment-Blanching

Thermal blanching is often used before drying. Its primary goals are to deactivate enzymes that cause spoilage in fresh raw plant material, reduce the microbial load to improve preservation, soften tissues for easier drying, and eliminate intracellular air to prevent oxidation [30]. Blanching involves rapidly heating plant tissue and holding it at a specified temperature for 1 to 10 min. Traditional blanching methods can be applied before drying to reduce the time required for dehydration [46].

Hot water blanching involves immersing the material in hot water at temperatures between 70 °C and 100 °C. After this process, the samples need to be cooled down. While essential water-soluble nutrient components may be leached out during blanching, the main concern is the high water consumption associated with the process [46].

Hot water blanching adversely affected the texture and microstructure of the sample, contributing to a greater loss of soluble nutrients and even extending the drying time [30]. However, hot-water blanching can also shorten the drying time. Domyaz et al. [47] observed a 14% reduction in drying time and an improvement in rehydration ratio by pre-treating quince by immersing it in hot water at 80 °C for 1 min. Hot water blanching of mushrooms reduced drying time by 5.78% for air-drying and 12.81% for electrohydrodynamic drying. Also, the antioxidant activity of mushroom slices treated with PEF and US was notably less than that of the slices that were blanched [48].

Steam blanching employs superheated steam that condenses on the food’s surface, delivering significant latent heat to the product. This method helps preserve most of the water-soluble minerals and nutrients, unlike water blanching. However, some irreversible changes in quality, particularly softening of the tissue, can occur [46]. Steam blanching significantly accelerated the drying of paprika pericarp by destroying cell walls, thereby reducing resistance to moisture transfer during drying. The steam blanching treatment also resulted in better vitamin retention due to the low-oxygen atmosphere [49]. Steam blanching pre-treatment of yacon resulted in faster hot-air drying than observed for samples without blanching. The blanching also reduced the concentration of inulin, glucose and fructose in dried yacon [50].

The choice to blanch before drying should be guided by empirical investigation, as different foods react uniquely. Investigations will clarify how blanching affects drying and ensure the final product meets quality standards [51].

### 2.3. Physicochemical and Chemical Treatment

The chemical pre-treatment process involves immersion in solutions containing osmotic agents, alkalis, acids, or sulphur-based compounds. The solutions used can lead to the partial dissolution of skin components—particularly lipids—as well as the chemical denaturation and rupture of the cell membrane, resulting from the osmotic pressure difference between its interior and exterior. These phenomena reduce the resistance to water diffusion toward the material’s surface. The choice of pre-treatment solution is determined by the material’s specific characteristics and the intended application. The properties of the plant tissue should guide the choice of chemical additives for immersion processes. Using strong chemicals can harm the delicate structure of fruits. Moreover, immersing vegetables in an osmotic sugar solution can change their flavour, which is often deemed unacceptable.

Osmotic dehydration is a method used before drying fruits and vegetables. In this process, the produce is immersed in a hypertonic solution at a controlled temperature. A counter-current, three-directional mass exchange occurs: water moves from the plant material into the solution, while dissolved substances flow from the solution into the products. Additionally, there is a slight diffusion of certain water-soluble substances, such as minerals, vitamins, sugars and acids, from the tissue into the solution. This exchange continues until the solute concentrations and osmotic pressures in both the solution and the material equalise; at this point, no further mass exchange occurs [52]. The main advantage of this pre-treatment is a 10–70% reduction in water content without phase changes, making the technique energy-efficient [53]. The application of osmotic pre-treatment can significantly reduce the duration of subsequent drying processes. The shorter drying time reduces the thermal treatment duration, thereby better preserving colours, nutrients, and bioactive compounds [52].

The type and rate of changes in tissue subjected to osmotic treatment depend on the process conditions, including the concentration of the osmotic agent, the process temperature, the solution-to-solid mass ratio, the size and geometry of the material, and the intensity of agitation of the solution [53]. Also, the efficiency of osmosis can be affected by the type of tissue, its composition and structure. Osmotic dehydration involves various substances, such as sugars, salts, and sugar alcohols, which can significantly influence the process [30]. Many studies have shown that osmotic pre-treatment accelerated the drying process. The drying time of melon subjected to osmotic dehydration in sugar solution at concentrations of 45 and 60°Brix was decreased by 23–46% in comparison to untreated samples [54]. A 35–65% decrease in drying time was also reported for microwave-freeze-dried potato pre-treated with salt and sucrose solutions at different concentrations. Additionally, an increase in hardness and crispness was observed in osmo-dried potatoes [55]. Sometimes osmotic treatment had the opposite effect. A decrease in drying rate was observed in osmo-dehydrated cashew apple in 52% sucrose or corn solutions. This pre-treatment also caused a great loss of vitamin C [56]. However, some studies showed that carrot slices soaked in a salt-sugar solution and air-dried received higher organoleptic scores for colour and odour than the control sample [57]. Also, osmotic dehydration of pineapples in a sugar solution after microwave–vacuum drying resulted in better colour retention, softer texture, improved rehydration capacity and lower shrinkage than in untreated dried fruit [58].

Osmotic treatment is a valuable technique for improving the drying process and the quality of the final dried products. However, it can also introduce undesirable characteristics or extend the drying time. Therefore, it is important to select the osmotic solution, concentration, and dehydration temperature based on the specific type of raw material and the drying method being used.

Sulfurization is frequently applied during the processing of fruits and vegetables. The application is mainly linked to the antimicrobial, anti-browning, and antioxidant properties of these sulphur compounds [23]. Mainly, sulphur dioxide gas or water-soluble sulphides, and salts such as potassium metabisulfite, sodium metabisulfite, and sodium hydrogen sulphite, are used in this pre-treatment. Sulphite ions function as an inhibitor of polyphenol oxidase (PPO). This occurs via a reaction between sulphite and quinones generated by PPO [30]. Sulfiting may facilitate drying by altering membrane permeability through modification of pectin polysaccharides. Alterations in tissue structure and the cleavage of disulfide bonds in proteins can also soften plant material [59]. Application of this treatment for an apricot reduced drying time by 3.6–38.6% and increased lightness compared to untreated dried samples [60]. However, sulfurization pre-treatment can also cause the loss of some water-soluble compounds, such as ascorbic acid, and their chemical residues can lead to many health problems. Therefore, the quantity of ingredients in the food product must be clearly stated on the label and should not exceed established limits [30].

Another chemical treatment is mainly applied to fruits like berries before drying to solubilise or destroy the outer wax layer. Some alkali liquors, including sodium carbonate, sodium hydroxide, and potassium carbonate, as well as oil emulsions (e.g., ethyl oleate), a mixture of oil with an alkaline agent, were used for this chemical pre-treatment [61]. Dipping grapes in 0.25% NaOH for a few seconds led to the formation of cracks on the fruit’s skin and a reduction in the convection drying time of raisins [62]. The same effect was observed when potassium carbonate and ethyl oleate solutions were applied before air-drying grapes. Drying rates were higher for treated grapes than for controls, and also, fruits dipped for a longer time dried faster. However, browning occurred in all dried-treated grapes [63]. A potassium carbonate–olive oil mixture was applied before drying the grapes, resulting in reduced drying time and increased lightness of the final product [64]. Alkali-dipping pre-treatment can cause degradation and oxidation of ascorbic acid due to the alkaline environment and surface microcracks [30].

Citric acid, as an organic acid, is primarily utilised as a preventive against darkening and a texture enhancer for plant tissues before drying. The application of this acid treatment altered the pectin structure and accelerated the drying process [30]. Treatment with citric acid or ascorbic acid reduced drying time and enhanced fruit colour. However, prolonged immersion in an acidic solution can degrade water-soluble nutrients [30,60].

Traditional pre-treatment methods have been shown to reduce drying times and may improve certain material quality properties. However, the application of these methods can also result in the absorption of chemical substances, increased energy consumption, alterations in the materials’ structural and textural properties, a decrease in nutritional value, and impaired rehydration capacity [65].

## 3. Non-Conventional Processing Methods Before Drying

Modern processing emphasises designing product quality. Depending on the desired attributes of the dried product, appropriate preparatory steps and drying techniques should be selected. Understanding the material’s characteristics and how each operation influences them is essential for optimal results.

Various biological characteristics complicate the drying process for fruits, vegetables, and meat. Key factors include the presence of cell membranes and, in the case of berries, the integrity of their skin. Certain fruits, such as raspberries, have a delicate structure that makes them unsuitable for cutting before drying. Many valuable nutritional compounds present in fruits and vegetables are thermosensitive and susceptible to oxidation [66]. Consequently, pre-treatment methodologies must enhance the permeability of biological membranes and skin structures. These processes should be performed at reduced temperatures to slow chemical and biochemical reaction rates, and they must occur under strictly controlled conditions to prevent leakage of intracellular fluids or their constituents. To enhance food preservation and quality, consider utilising advanced processing techniques such as microwave blanching, steam blanching under elevated pressure, high-pressure processing, ohmic heating, pulsed electric field treatment, ultrasound treatment, and infrared blanching. Immersion in calcium chloride (CaCl_2_) or ethanol solutions is also effective in maintaining nutritional and sensory attributes.

When drying leafy vegetables or herbs, ensuring the microbiological purity of the dried products is a significant concern. Therefore, it is advisable to implement specific operations before drying to reduce microbiological contamination. UV-C radiation, infrared radiation, or cold plasma are recommended for this purpose. Ozonation or a carbon dioxide atmosphere can also be used.

Berries pose distinct challenges for the drying process, primarily due to their delicate and permeable skins. This characteristic complicates moisture removal and can lead to suboptimal drying outcomes. To address these challenges, various methods can be employed, including mechanical, chemical, or enzymatic treatments to enhance skin permeability. The application of specific enzymes can weaken or disrupt the integrity of berry skins, facilitating more efficient moisture evaporation during the drying process.

### 3.1. Microwave Blanching

**Figure 2:**
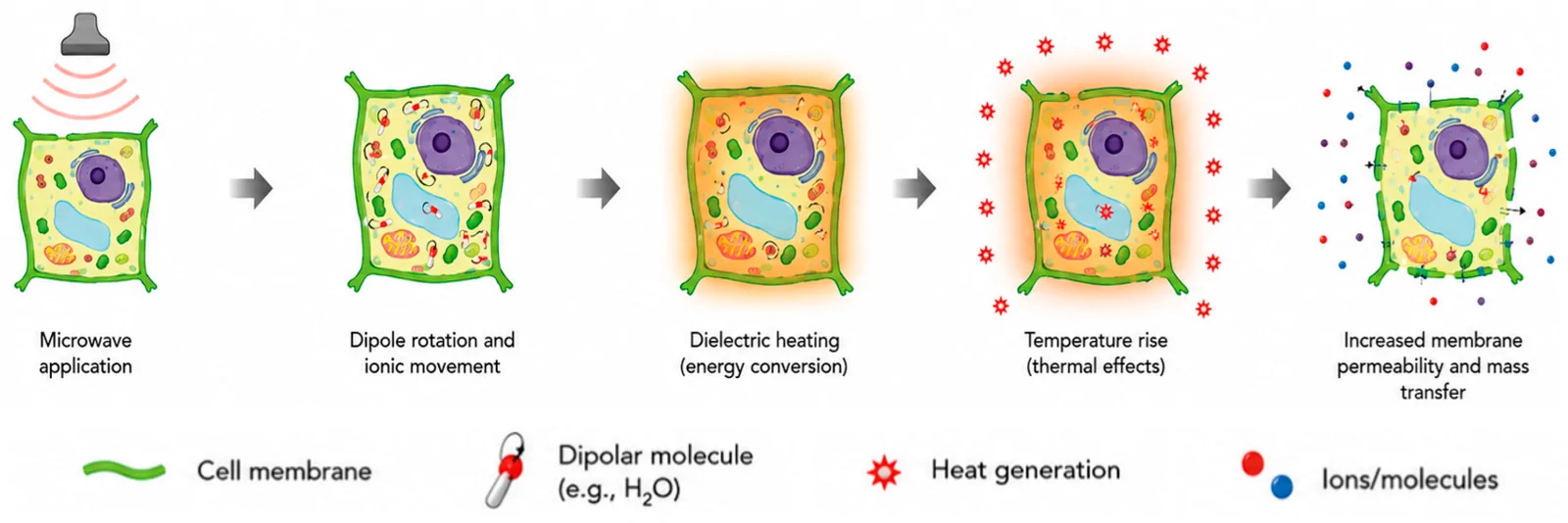
Effect of microwave heat on biological tissue.

Microwave blanching uses electromagnetic waves with frequencies ranging from 300 MHz to 300 GHz. When food raw materials, characterised by high water content, undergo a microwave treatment, the extensive presence of water dipoles facilitates their rotation. This process generates friction, which contributes to the disruption of hydrogen bonds. As these dipoles rotate in response to applied heat, the resulting energy weakens intermolecular forces, ultimately affecting the material’s physical and chemical properties. At the same time, charged ions move, which contributes to energy generation and heat production. The heat generated within the material causes thermal degradation of cell membranes in both tissues and microorganisms, as well as enzyme inactivation (Figure 2). In traditional blanching, heat transfer primarily occurs via convection and conduction, resulting in longer processing times and large temperature gradients within the heated material. Therefore, thermal denaturation of cell membranes or enzymes occurs in the layers closer to the surface. In contrast, microwave heating uniformly heats the material, resulting in faster processing [30,67].

Microwave blanching is typically used to inactivate enzymes and reduce microbial activity in fruits and vegetables. The application of microwave blanching, which reduces drying time, improves efficiency, enhances nutrient retention, and maintains the porosity of dried materials compared with hot-water blanching pre-treatment. Microwave blanching reduced the drying time of mushroom slices by 22% compared with hot-water blanching. Additionally, the microstructure of the MWB-treated material was more consistent than that of the material obtained by water blanching [68]. MWB resulted in the quick inactivation of peroxidase and minimised drying time, while also producing the highest levels of anthocyanins following microwave-assisted spotted bed drying of purple sweet potato. However, steam and hot-water blanching were found to improve the retention of purple colour in the potato after drying. This may result from a lack of air (oxygen) access to the material surface, which is surrounded by water or water vapour [69]. The drying rate of tomatoes subjected to microwave blanching at 100 W exceeded that of tomatoes pre-treated with hot-water blanching. Furthermore, the retention levels of ascorbic acid and lycopene, as well as the preservation of colour quality, were notably greater than those of the boiling water treatment [70]. The application of microwave pre-treatment accelerated mass transfer and increased energy efficiency during air-drying of white yams. A notable observation from the study is that as the pre-treatment duration increased from 1 to 5 min, the drying time decreased significantly, from 630 min to 390 min at an air temperature of 70 °C. Furthermore, under identical drying conditions, the effective moisture diffusivity of microwave-treated samples increased substantially, from 1.05 × 10^−8^ to 1.57 × 10^−6^ m^2^/s. In contrast, dried samples subjected to hot-water pre-treatment showed a decline in effective moisture diffusivity from 1.02 × 10^−8^ to 8.81 × 10^−9^ m^2^/s as the blanching time increased. The process of water blanching results in significant starch gelatinisation, which modifies tissue structure and increases viscosity, thereby increasing resistance to moisture transfer. Both pre-treatments adversely affected ascorbic acid levels and the occurrence of nonenzymatic browning. Microwave pre-treatment resulted in a smaller reduction in ascorbic acid content in dried yams compared to the conventional water blanching method [71].

Well-controlled pre-treatment with microwave energy can increase blanching efficiency and retain bioactive compounds and sensorial attributes in many plant materials. Process parameters, such as blanching time and applied microwave power, are crucial for maintaining product quality. Longer blanching times (2 to 8 min) and higher microwave power (150 to 600 W) led to darker, softer green asparagus with lower phenolic concentrations [72]. Microwave blanching at 720 W for 2 min and at 900 W for 5 min was employed before various drying methods for okra. Specifically, in the context of tray drying at 60 °C, increasing microwave pre-treatment power output reduced the drying duration from 540 min to 330 min. However, this reduction in drying time was accompanied by an increase in the lightness (L*) of the dried samples. Furthermore, the effect of enhanced microwave energy during the blanching process was similarly manifested in the combination of microwave and tray drying methods. Microwave-assisted blanching at higher power and hybrid drying at 70 °C reduced the drying time to 210 min while preserving the highest greenness index [73].

The application of microwave blanching, rather than traditional blanching methods, maintains colour and nutrients and reduces drying time more effectively; however, it is essential to use the appropriate power levels to prevent a decline in quality during processing. High microwave power and longer blanching time may lead to overheating or charging of the material, as well as deterioration of its quality. Better retention of flavonoids and anthocyanins was observed for freeze-dried cranberries with a microwave pre-treatment at 100 W than at 500 or 800 W. Higher microwave energy may alter texture and structure, compromise tissue integrity, and release many important bioactive ingredients [74].

Although microwave blanching offers numerous advantages, some drawbacks may restrict its use. One significant challenge is controlling the process, as the treatment is often inconsistent due to non-uniform electromagnetic fields, particularly in terms of temperature. This difficulty may stem from frequency-dependent factors, such as the size and composition of the biological material samples. Additionally, the limited penetration depth of microwaves can impede the uniform distribution of energy throughout the blanched materials [30].

### 3.2. Superheated Steam Impingement Blanching

Superheated steam impingement blanching (SSIB), also known as high-humidity hot-air impingement blanching (HHAIB), is a thermal technology that combines superheated steam blanching and impingement. Elevated thermal attributes, including enthalpy, heat transfer coefficient, and heat transfer rate, can characterise HHAIB. The enhanced heat-transfer rate during processing facilitates rapid, uniform heating of food products, thereby preserving essential nutrients while optimising energy efficiency [30,61].

High-velocity steam impingement effectively eliminates the water boundary layer on the product’s surface. The high-velocity air from the nozzles of the impingement system can generate a cushion of air that suspends the products [75]. With superheated steam impingement, the thermal boundary layer is thinner, which improves heat transfer. The HHAIB or SSIB process consists of two stages. First, steam condenses on the product’s surface because the product’s temperature is below the saturation temperature of the steam. This condensation transfers latent heat to the sample. Second, as the material’s temperature rises, a significant amount of water evaporates from the product into the steam [76].

Bai et al. [77] applied HHAIB at temperatures ranging from 90 to 120 °C and for treatment durations of 30 to 120 s, followed by air-drying. The application of HHAIB reduced drying time and resulted in high-quality dried raisins. Drying time was reduced by about 42% when the blanching time was increased to 120 s. During the consistent treatment duration, an increase in blanching temperature within the examined range reduced drying time by 11%. During HHAIB treatment of grapes, an increase in internal pressure above ambient levels causes micro-cracks to form on the grape peel. The development of these microstructural features enhances the permeability of the fruit’s membranes and softens the tissue. This, in turn, facilitates water diffusion and speeds up the air-drying process. Additionally, the application of HHAIB may inhibit enzymatic browning by decreasing polyphenol oxidase (PPO) activity [78]. The residual activity of polyphenol oxidase (PPO) in dried grapes declined as blanching time and temperature increased, reaching a maximum reduction of approximately 8.74% [77]. Also, Xiao et al. [79] noticed that pre-treatment with SSIB enhanced drying and increased the whiteness index of yam slices, likely due to the lack of oxygen. The use of super steam impingement blanching (SSIB) before air-drying red pepper reduced the drying time by up to 4 h compared with untreated samples. The peppers that underwent SSIB treatment showed higher levels of red pigments, better retention of ascorbic acid, increased total antioxidant activity, and higher DPPH values compared with those that were blanched in hot water [80].

The application of superheated steam impingement blanching has been shown to enhance the drying process while minimising adverse alterations in product quality. Despite its advantages, the industrial application of this method remains constrained by high operational costs, complex equipment design, and challenges in achieving uniform heating throughout the product [30,76]. Additionally, in some products, undesirable changes in texture may occur; for example, excessive starch gelatinisation can hinder the drying process [79]. The SSIB process should be optimised and controlled for each food type to prevent negative effects from changes in material texture and yield loss.

### 3.3. High-Pressure Processing

High-pressure processing (HPP) is primarily used for fruits and vegetables, but also for seafood and meat. High-pressure processing involves compressing a medium to a specified pressure, maintaining this pressure for a specified time, and then decompressing it. This pressure is transferred using a suitable fluid (water, glycol, ethanol, sodium benzoate, silicone oil), and the product is placed in flexible packaging. Both pressure increase and decompression can occur at different rates of pressure change.

When conducting HPP, the following process parameters should be considered: temperature, time required to achieve the desired pressure, pressure height and duration, decompression time, pH, and water activity. A broad range of pressure values is utilised in studies, varying from 20 MPa to 1000 MPa. Temperatures can range from 20 °C to 105 °C, and the duration of pressure exposure can span from fractions of a second to several hours. Additionally, the number of pressure pulses can vary from a few to several dozen [81]. When the objective of processing involves the disintegration of plant or animal tissue cells, the temperature generally remains below 50 °C [82]. The application of elevated temperatures is a method for eliminating microbial flora.

HPP conditions should be selected individually for each material. HPP is a relatively new technique used primarily as a non-invasive method for inactivating microorganisms in food [83]. The mechanism of action involves the destruction of cell membranes. This destruction occurs primarily during the decompression stage. During the period of maintaining a given pressure, the pressure inside and outside the cell is equalised. During decompression, the presence of cell membranes causes decompression to occur primarily outside the cell. This creates a pressure difference between the inside and outside of the cell, leading to cell rupture. For example, at 400 MPa HPP, red peppers yielded a cell disintegration index of 0.58. An increase in membrane permeability increases the mass transfer coefficient [84]. Cellular fluid leaks from damaged cells, increasing water mobility. This phenomenon can be observed using low-field nuclear magnetic resonance (LF-NMR). The diffusion coefficient of water in the material also increases, up to threefold [85]. As a result, drying time was shortened by up to 40% [86,87,88]. Water mobility increases with increasing applied pressure. As the pressure applied during processing increases, the fruit’s porosity and pore size increase.

Low-molecular-weight compounds, including flavour compounds, pigments, and biologically active molecules such as vitamins, remain intact during HPP treatment. However, changes in the structure of other components, such as proteins and enzymes, may occur. These phenomena may, in some cases, limit this method’s usefulness for gentle food processing. In contrast, in others, they may be beneficial for shaping the desired properties of fruit products [89].

After HPP treatment, pectin and polysaccharides are modified, resulting in a looser structure and reduced mass-transfer resistance [86]. The increase in soluble pectin content was attributed to the β-eliminative depolymerisation of matrix polysaccharides at high pressure [90]. Zhang et al. [86] also observed a more than two-fold increase in total anthocyanin content after HPP treatment. HPP treatment occurs without high temperatures; therefore, heat-labile compounds are preserved. It has a positive effect on antioxidant activity, reducing its loss during drying [85]. Pre-treatment with HPP can improve the flavour and aroma of dried fruits. Among other things, an effect on the volatile and ester compounds in freeze-dried strawberries was observed, resulting in increased floral, fruity, and sweet notes. Greater sweetness and lower acidity were achieved [91]. The effect of treatment on the texture of dried fruits is not clear. HPP treatment may reduce texture parameters such as hardness, elasticity, and chewiness in pineapple [88], but it had a positive effect on the texture of Angelica keisei FD slices [87]. The effect of high pressure on the texture of plant materials can be linked to the material’s structure and texture before pre-treatment. More cohesive, firmer raw materials may respond differently to HPP than softer materials. In carrots, both hardness and chewiness diminished at 400 MPa, but no further reduction was observed at pressures up to 1200 MPa, indicating a threshold effect related to the initial structure. Furthermore, high-pressure processing can induce changes in the structure of pectin, such as depolymerisation, the solubilization of water-insoluble pectin, and the interconversion of different fractions. The texture of plant tissue is affected by changes in cell structure, membrane stability, and pectin characteristics due to applied pressure [92]. In high-protein foods, such as meat and seafood, high-pressure processing can alter protein structure, leading to either enhanced or degraded texture [93]. The protein’s original characteristics and its response to applied pressure are key factors in forecasting texture modifications. Applying moderate pressure (100–400 MPa) caused cell disruption and softening of carrot tissue. However, at elevated pressures (over 1000 MPa), cohesiveness was restored as structural components began to reform [92]. In the case of apples, high pressure (300–400 MPa) caused significant leakage from the tissue and a decrease in firmness [94]. Application of lower temperature 0–35 °C with HPP minimised negative changes in the texture of dry-cured ham [93]. The impact of HPP on food texture depends on the pressure level, application duration, and temperature. The effects of pressure and temperature vary by material; certain combinations can be advantageous for some products, while others may degrade [95]. HPP treatment may also affect the sorption properties of dried fruits, shifting the equilibrium water content for a given water activity toward higher values [85]. HPP also positively affects the ability to rehydrate after drying [96].

HPP is also used to destroy microorganisms and inactivate enzymes. Microorganisms differ in their responses to HPP: yeast and vegetative moulds are generally relatively sensitive to pressure, but responses depend on individual characteristics. Sometimes, there are substantial differences in pressure resistance, even among individual strains of the same bacterium [97]. In the case of yeast inactivation, it occurs at milder conditions than for bacteria, due to cell size. Depending on the microorganism, reductions of up to 8 logarithmic cycles are achieved.

An extension of the HPP method is the multipulse (mpHPP) treatment. This involves multiple cycles of compression and decompression [81]. The effectiveness of the mpHPP treatment depends on selecting appropriate pressure, initial and target temperatures, pulse number, pulse holding time, and compression and decompression. mpHPP can be used to inactivate microorganisms in food at lower pressures than single-pulse processing. Recent advancements in knowledge and technology have enhanced high-hydrostatic-pressure processing by optimising compression and decompression rates. This leads to consistent high-pressure pulses, which improve overall processing efficiency [81]. Furthermore, these advancements improve rehydration after drying, thereby enhancing the effectiveness of rehydration [96].

### 3.4. Ohmic Heating

Ohmic heating is a process in which heat is generated by the flow of alternating current through a food material. The ohmic heating system consists of a heating chamber, two electrodes, an alternating-current power source, and units for controlling the voltage and measuring the temperature. It also includes a variable transformer and a recording and display unit. Foods typically contain a large amount of water and dissolved salts, which provide ions that move to electrodes of opposite charge in the electric field. This movement leads to collisions between ions and friction with stationary matrix particles, producing heat in the material. Ohmic heating is affected by the electrical conductivity of foods, which depends on temperature, bond dissociation, the intensity and frequency of the applied electric field, heat capacity, and the electrode material. The composition of materials (water content, ion concentrations) and their structure can affect electrical conductivity. Heat is transferred volumetrically in solid and liquid foods at the same rate, regardless of the product’s shape or size [30,98,99]. The interplay between electroporation and the softening of the tissue matrix induced by alternating current likely elucidates the underlying mechanism of ohmic heating. Three mechanisms can describe moisture diffusion during ohmic heating. Increased internal diffusion, as well as increased vapour pressure, leads to volumetric heating. Non-uniform electrical conductivity led to the generation of internal heat and a vapour-pressure gradient. These effects create an ongoing pathway that allows moisture to move from the material’s inner core to its outer layer, where it is released into the surrounding environment. This mechanism prevents the formation of a hard layer on the material’s surface, thereby facilitating moisture transfer [100].

Applying this pre-treatment technology before drying reduces drying time, retains many bioactive compounds, and preserves colour and other nutrients in foods [99]. Additionally, it maintained the quality and stability of dehydrated food items during storage [100]. Ohmic heating can be used as a more efficient, faster, and more environmentally friendly blanching technique than conventional thermal methods used before drying [30]. Ohmic heating conditions, such as voltage, frequency, current density, and process duration, may affect the drying process, especially the rate of moisture removal and energy consumption. Many studies have shown that higher electric field strength increases the drying rate. Lebovka et al. [101] observed that ohmic pre-treatment at low fields (<100 V/cm) led to incomplete damage of potato tissue. However, the electric field and total electric energy input affected the drying process. Increasing the electric field E from 60 to 100 V/cm increased the drying rate, but the drying process was further accelerated by increasing the energy input from 20 to 100 kJ/kg at the same E = 100 V/cm. The pineapple cubes treated with an electric field strength (EFS) of 25 V/cm showed a reduction in drying time compared to untreated samples. The drying time decreased from 240 to 180 min as EFS increased from 25 to 35 V/cm. Faster drying at higher EPS was associated with more severe tissue damage and cracks in the material’s skin. Ohmic pre-treatment before drinking also had a positive effect on the hydration capacity. However, increasing the ohmic treatment time did not affect the rehydration ratio of the pineapple cubes [102]. The ohmic pre-treatment reduced the drying rate of hot air-dried tomato paste by 80–90% compared to untreated samples. The increased voltage gradient from 6 to 16 V/cm led to shortening of drying time [103]. Different ohmic heating frequencies can affect the drying process. Ohmic pre-treated grapes dried significantly faster than untreated grapes, with the drying rate highest at low frequencies (30 and 60 Hz) and lowest at high frequencies (7.5 kHz) [104]. Ohmic heating can reduce energy consumption by shortening drying time compared to conventional methods. The increased heat energy input at higher voltages results in higher energy consumption [100].

Ohmic heating before drying can better maintain colour and sensory properties than conventional techniques. Ohmic pre-treatment, conducted at an electric field strength of 16 V/cm, resulted in greater increases in total phenolic content, antioxidant capacity, and red pigment concentration in thin-layer dried red pepper than both hot-water and microwave blanching [105]. Ohmic treatment of litchi caused changes in dried pulp colour. However, intermittent ohmic heating preserved colour better than intermittent air-drying. Increased lightness and reduced browning were observed in ohmically treated samples [106]. The colour of quince samples obtained with ohmic heating for osmotic dehydration pre-treatment, followed by microwave drying, was closer to that of the raw materials. Also, the rehydration ratio increased as the electric field strength decreased [107]. However, the intense ohmic heating may degrade pigments. The increased colour changes in pomegranate material due to electrochemical and chemical reactions were observed at higher voltage gradients of 7.5 and 12.5 V/cm [108]. Increasing the electric field from 20 to 40 V/cm before drying purple-fleshed potato slices resulted in higher total phenolic content and increased anthocyanin levels [109]. The highest total phenolic content was also observed in the ohmic-treated microwave-dried quince than in the control material [107]. The oxidation of vitamin C was affected by the duration and temperature of ohmic heating. Extended duration and reduced rate of ohmic heating treatments resulted in a significant decrease in ascorbic acid concentrations within dried materials [100].

The main drawback of using ohmic heating as a pre-treatment method is the difficulty of controlling the temperature of complex materials. Furthermore, hydrogen and oxygen generated by water electrolysis may result in a decline in quality [30].

### 3.5. Pulsed Electric Fields

**Figure 3:**
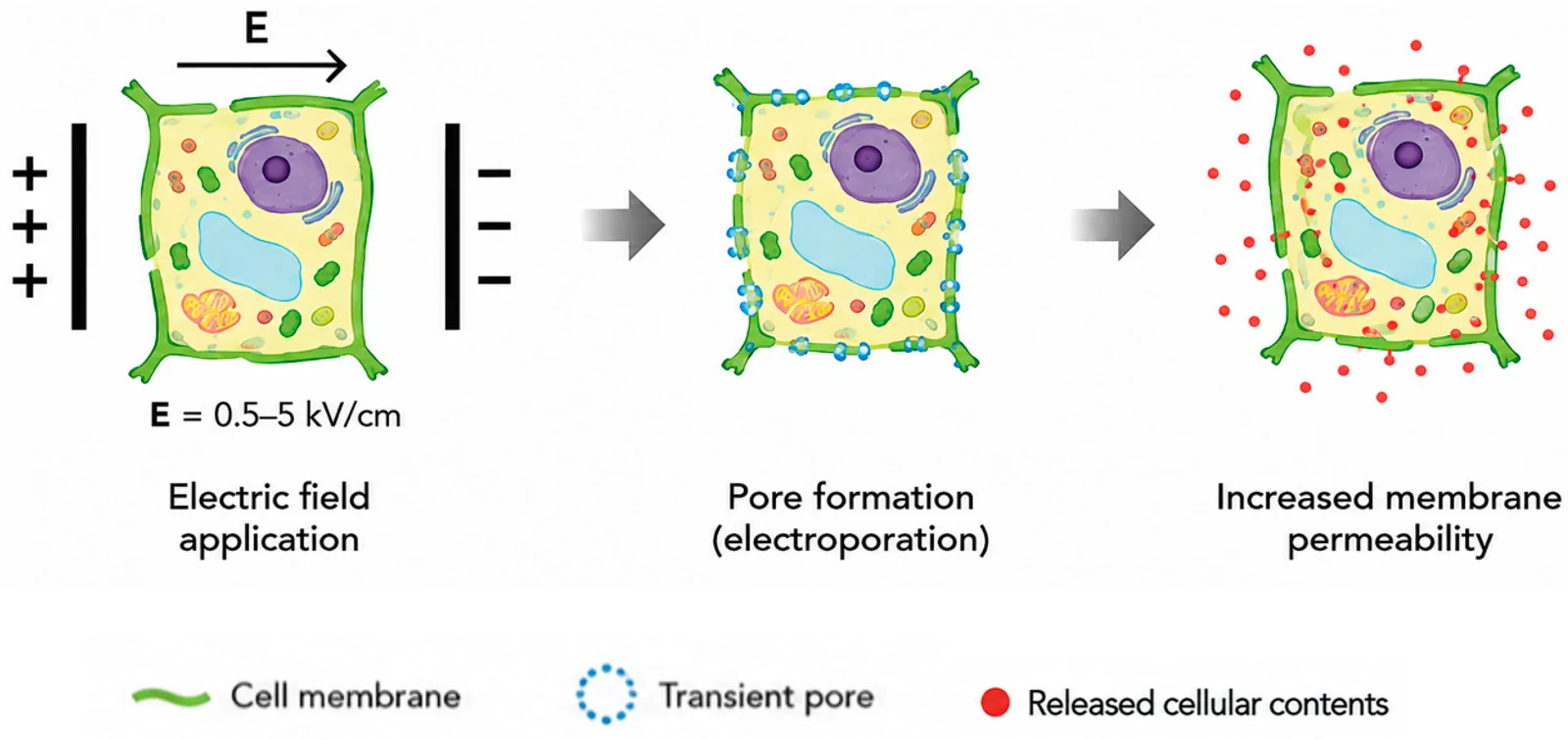
Effect of pulsed electric fields on biological tissue.

Pulsed Electric Field (PEF) technology uses short-duration electrical pulses that can alter the structure of cell membranes (Figure 3). These pulses generate temporary or permanent pores that can reduce the resistance to mass transfer of substances contained within the cell and facilitate mass transfer. Cell electrofusion is routinely used in cell biology to transfer proteins, RNA, or DNA [110]. The detailed mechanism of this technology has not been fully understood [111].

A cell can be described as a system consisting of three regions: the cell membrane, intracellular fluids, and extracellular fluids, each with specific electrical conductivity determined by its chemical composition. Under the influence of an external electric field, such a system behaves like a spherical capacitor: the cell membrane, which is most often an insulator, separates the cytoplasm, which has a specific electrical conductivity, from the external solution, which serves as a conductive buffer. Kinetic studies of electropermeabilisation led to the description of a phenomenon occurring in 5 steps [110]:

- Induction—the field caused an increase in the membrane potential difference; when it reaches a critical value (approximately 200 mV), local discontinuities (defects) appear, which may be caused by kinks in the lipid chains; mechanical stress occurs, the magnitude of which depends on the buffer composition.
- Expansion—this phenomenon occurs when an electric field exceeds its critical value, causing electromechanical stress.
- Stabilisation—once the field intensity falls below the critical value, the membrane becomes permeabilised to small molecules; the stabilisation process occurs within a few milliseconds.
- Sealing—a slow, resealing occurred, occurring over seconds and minutes.
- Memory—some changes in membrane properties persist over a longer time scale (hours), but cell behaviour eventually returns to normal.

A key limitation in understanding electropermeabilization is the lack of information on membrane transitions that facilitate the transmembrane transport of polar compounds.

From the above, it follows that the effect of the applied electric field and the magnitude of the electromechanical stress depend on the electrical conductivity of the fluids inside and outside the cell. Additionally, the cell membrane can exhibit low electrical conductivity. Therefore, the effect of pervaporation at a given external field voltage may vary across different biological materials and depend on the applied voltage. Depending on the processing parameters, varying degrees of tissue degradation can be achieved. The parameters that need to be designed are: electric field value E (kV/ cm), medium temperature (°C), frequency of the applied electric field (Hz), number of pulses (n), pulse width (μs), and process time (s) [112]. The material is placed between two electrodes, with an electric field strength of 0.5–100 kV/cm and a pulse duration of microseconds–nanoseconds [113]. Depending on the electric field strength, one can achieve:

- Reversible electroporation (0.5–1.5 kV/cm, 0.5–5 kJ/kg) [114];
- Wnhancing mass transfer in plant or animal cells (0.7–3.0 kV/cm, 1–20 kJ/kg) [2];
- Sludge decomposition (10–20 kV/cm, 50–200 kJ/kg) [115];
- Microbial inactivation (15–40 kV/cm, 1000 kJ/kg [116].

Critical values of the applied electric field for plants and microorganisms are in the range of 1–2 kV cm-1 and 10–14 kV /cm (e.g., *Escherichia coli*), respectively [117]. Higher electric field intensity (e.g., 0.25–5 kV/cm) and higher pulse energy lead to more severe damage to cell membranes. As a result, cell membrane permeability and mass transfer efficiency increase. By increasing the applied energy, we can achieve both reversible and irreversible pervaporation. A greater number of pulses and a lower frequency (less than 1 Hz) cause greater tissue damage than higher frequencies, as explained by the cumulative effects of cellular changes and cytoplasmic movement [118].

Short, gentle treatments can lead to controlled permeabilisation without significant degradation, whereas intense, long-lasting pulses can cause significant morphological changes (loss of turgor, softening, and sap leakage [119]. Excessive energy can cause irreversible damage to the tissue structure, e.g., cell disintegration, degradation of bioactive compounds, and loss of firmness.

Tissues with larger cells are more susceptible to damage, but there is not always a simple correlation—the material’s electrical conductivity and degree of hydration are also important. Tissues with a high ratio of electrical conductivity between intact and damaged tissues require higher field strengths to achieve a similar effect.

Ben Ammar et al. [120], examining apple, potato, carrot, courgette, orange, and banana, found that the optimal electric field strength was an increasing function of the ratio of the electrical conductivities of intact and damaged tissues. Plant tissues with a high ratio of electrical conductivity between intact and damaged tissues required a rather strong field. Therefore, the response of raw materials to PEF depends on their structure, composition, and conductivity. Each parameter selection should be preceded by an assessment of the technological goal and the raw material characteristics, including measurements of indicators such as electrical conductivity, hardness, and colour that reflect the degree of degradation. From a practical perspective, the potential of PEF treatment lies primarily in its ability to shorten drying time significantly. To achieve this, irreversible electroporation is necessary, as it alone can reduce mass transport resistance.

PEF pre-treatment of kiwifruit significantly shortened the drying time without affecting final product quality [121]. PEF pre-treatment reduced drying time by 27% for potatoes [122], 30% for onions [54], and even 57% for basil [123]. However, it is worth noting additional benefits, such as increased microbiological purity [124], including reductions in spore counts [125] and aflatoxin levels [126]. An increase in the extraction efficiency of biologically active ingredients was also achieved. Belgheisi et al. [127] demonstrated that applying 30 PEF pulses at 0.25 kV/cm (0.6 ms) can increase lycopene extraction by 88% and effectively maintain its antioxidant activity. Rybak et al. [128] reported that PEF can induce the formation of free radicals and reactive oxygen species, thereby reducing antioxidant activity. However, shortening the drying time reduced the dried material’s exposure to oxygen. It can also partially inhibit oxidative enzymes. Negative aspects of PEF application include deterioration of texture, unfavourable colour changes, and degradation of biologically active components. Some of these are biological in nature (e.g., enzymatic browning as a cellular response to cell membrane damage), while others may result from improperly selected parameters. PEF technology has low operating costs and can reduce processing steps. It allows for the production of high-quality products. However, the high initial cost is the most significant barrier to its industrial application. To better leverage the method’s potential, it is worthwhile to create databases of metabolic pathways and nutrient retention in fruits and vegetables that correspond to the electric-field effect [21].

### 3.6. Ultrasounds

**Figure 4:**
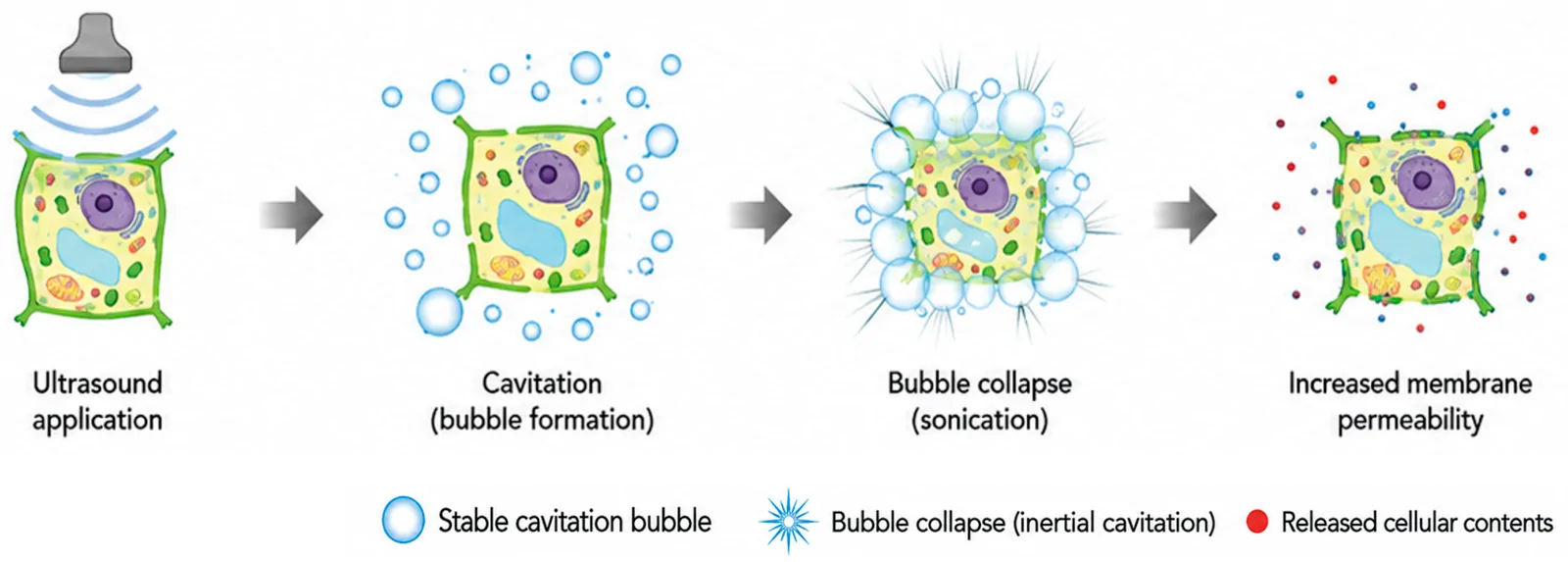
Effect of ultrasound application on biological tissue.

Ultrasound is a type of mechanical wave with frequencies ranging from 20 kHz to 1 MHz. The range of 20 to 100 kHz is called low-frequency ultrasound, corresponding to wavelengths in water between 7.4 cm and 1.5 cm. Intermediate frequencies (100 kHz–1 MHz) correspond to wavelengths of approximately 15–1.5 mm in water, and high frequencies (1–10 MHz) correspond to wavelengths down to approximately 150 μm in water. Pre-treatment involves applying ultrasound using an ultrasonic bath or probe that emits ultrasonic waves into an aqueous medium, most often distilled water. In a gaseous medium, acoustic energy is significantly attenuated, limiting its application. These waves travel within the material or on its surface at a speed characteristic of the emitted wave and dependent on the material in which they propagate (chemical composition, structure, texture). Acoustic waves cause local compression and expansion of the material, producing a sponge-like effect. As a result of these deformations, microscopic channels form in the cell membrane, through which intracellular fluid can leak into the surrounding environment (Figure 4). When the ultrasound power is sufficiently high, rapid evaporation of liquid molecules (cavitation) can occur during expansion, forming cavitation bubbles. These bubbles grow and, once they exceed a critical size, burst violently. This causes rapid, high changes in local pressure and temperature [129,130]. The collapse of cavitation bubbles can generate powerful micro-jet streams that disrupt the laminar sublayer at the liquid–solid interface. This promotes heat and mass transfer [131].

It is possible to use ultrasound without water, but the sound energy must have good contact with the material it is meant to affect. When the vibrating probe is in direct contact with the food, effective acoustic impedance matching allows the ultrasonic energy to penetrate deeply into the raw material. This method is utilised in ultrasonic-assisted drying [129], although it is difficult to implement.

Pre-treatment with ultrasonic waveforms requires selecting appropriate parameters, such as ultrasonic power, sonication time, frequency, and sonicator probe amplitude. In numerous studies on the use of US as a pre-treatment before drying, researchers on a laboratory scale use frequencies ranging from 20 to 130 Hz, treatment times ranging from 1 to 60 min, US power ranging from 100 to 1500 W, and temperatures ranging from 20 to 30 °C [129]. Some studies use a dual-frequency, sequential or simultaneous mode, e.g., 20 Hz followed by 40 Hz or both 20 and 40 Hz [132]. If pre-treatment occurs in an aqueous environment, US exposure facilitates the efflux of cytoplasmic components from cells, thereby promoting the loss of soluble dry matter and water absorption. For this reason, it seems worthwhile to conduct research using isotonic solutions (relative to the raw material) as the ultrasound-transmitting medium to limit mass movement during processing [132]. The effect of treatment on drying rate does not always reduce drying time; the most frequently studied is ultrasound exposure time. Research on the effects of ultrasonic power, frequency, and amplitude is limited. Some studies report significant increases in drying rates; for instance, Andean blackberries treated with ultrasound for 20 min exhibited nearly a fivefold increase in drying rate and an almost twofold increase in effective diffusivity [133]. However, other authors did not find a positive effect of ultrasonic pre-treatment on apple drying kinetics [134]. This variability in the effects of ultrasonic pre-treatment on drying kinetics may be due to the complex interactions among drying conditions, food structure, and water gain or loss during ultrasonic processing. It may also result from the inappropriate selection of process parameters. Dehsheikh and Dinani [135] achieved a significant reduction in drying time (about 20%) at 1000 W. A power of 500 W produced beneficial changes, but to a much smaller extent (about 10%). In turn, Xu et al. [132] used a dual-frequency mode in either sequential or simultaneous mode, reducing strawberry freeze-drying time by 40% and 50%, respectively.

When evaluating the quality of dried fruit pre-treated with ultrasound (US), researchers primarily focus on several key characteristics: colour, rehydration properties, rheological properties, shrinkage, and porosity/density. The observed changes in these characteristics can vary significantly, largely depending on the type of material being treated and the ultrasound power used. Dehsheikh and Dinani [135] obtained a significant increase in banana porosity, ranging from 10 to 15%. Xu et al. [132] used sequential dual-frequency processing and obtained relatively large pore diameters in freeze-dried strawberries, demonstrating the effectiveness of cell disruption during processing. In dried fruits and vegetables, bioactive compounds such as phenols, flavonoids, carotenoids, and anthocyanins, along with antioxidant activity, are evaluated. Local changes in pressure and temperature can lead to the decomposition of water molecules into H+ and OH− free radicals. Their presence enables various chemical reactions, including the oxidation of bioactive compounds in food and other reactions that modify molecules present in the material [130]. The formation of free radicals does not support the preservation of bioactive compounds like phenols. However, it can enhance the antioxidant activity of other components, such as flavonoids. Depending on the process and the surrounding matrix, the chemical effects of acoustic cavitation can either be beneficial or detrimental [136]. In the case of bananas, a more than two-fold increase in phenol content was demonstrated, and this increase depended on the applied ultrasound power [135]. Ultrasonic pre-treatment did not positively affect the vitamin C content of freeze-dried strawberries [132]. Prolonged ultrasonic exposure can lead to a significant accumulation of free radicals in strawberries, thereby accelerating VC oxidation. Ultrasound treatment can reduce dissolved oxygen levels, thereby preventing the oxidative degradation of phenols. Ultrasound can support phenol extraction, leading to the release of phenolic compounds or the inactivation of phenol-degrading enzymes [137].

Research suggests that controlling ultrasound-induced sonochemical reactions is essential for the successful implementation of ultrasound technology in food processing, particularly through the introduction of radical scavengers. However, the sonochemical hydroxylation of phenolic compounds appears to be an effective method to enhance their antioxidant properties [136].

Ultrasound treatment can also reduce the microbial load in raw materials by disrupting microbial cell membranes and the double-helical structure of DNA [132].

In their review of studies on the use of ultrasound in food processing, the authors do not emphasise the creation of conditions that enhance ultrasound’s effectiveness. The effectiveness of ultrasound depends on frequency, power, temperature, transducer type, signal type, reactor geometry, and the physicochemical properties of the liquids and food products being processed. With increasing distance from the transducers, acoustic cavitation is attenuated, as evidenced by a decrease in ultrasound intensity [138].

Operating at low frequencies (20–100 kHz) leads to the formation of large bubbles that collapse with high intensity. Therefore, low-frequency operation is suitable for applications requiring strong sonophysical effects, such as cell disruption or biopolymer degradation. At frequencies higher than 100 Hz, the effect is milder and can be used to inactivate enzymes [139]. Frequencies above 500 Hz promote a general increase in the efficiency of free radical formation resulting from the sonolysis of water molecules. The optimal frequency depends primarily on the balance between chemical effects (radical formation) and physical effects (cell fragmentation and mass transfer) [140]. For most transducers, the amplitude can be adjusted from 20 to 100%.

Power is the rate at which acoustic energy is emitted over time. It is related to the pressure amplitude of the sound waves. It must be sufficiently high to provide the pressure required for cavitation. Increasing acoustic power increases the temperature, number, and size of cavitation bubbles, as well as the sonochemical efficiency.

Cavitation activity within a reactor shows significant variability, characterised by areas of intense activity alongside regions known as “dead zones.” The size and distribution of these dead zones depend on several factors, including the reactor’s specific design, the ultrasonic probe used, and the transducer type and configuration (tube or plate). Additionally, two important parameters often overlooked in the analysis of cavitation dynamics are the probe immersion depth and the overall liquid level in the reactor. These parameters significantly affect the reflection and refraction of acoustic waves, thereby influencing the distribution of cavitation activity. Positioning the probe near the bottom or base of the reactor enhances sound-wave reflection, thereby expanding the cavitation zones. In contrast, moving the probe farther away from the bottom reduces the strength of the acoustic waves. The design of the bottom—whether it is flat or spherical—significantly influences this attenuation. Additionally, it was observed that the concentration of •OH radicals at an immersion depth of 60 mm was seven times greater than at immersion depths of less than 30 mm [140]. For plate transducers, the liquid height should be set as an odd multiple of one-quarter wavelength, positioning the air–liquid interface at an antinodal point to enhance the amplitude of the standing-wave component. Cavitation activity is influenced by the ratio of the probe or transducer diameter to the treatment tank diameter [141].

Another important parameter to consider is the operating mode of the ultrasonic signal. Ultrasonic waves can be transmitted in various modalities, including continuous-wave, pulsed, and swept modes. Continuous operation may result in excessive bubble formation, leading to sound wave attenuation and reduced cavitation activity. Pulsed sonication offers several advantages, including lower temperature rise, decreased signal attenuation, and expanded active zones. Forced liquid flow through mixing or circulation can be beneficial, but excessive circulation is undesirable. The efficacy of ultrasound treatment depends on the medium’s viscosity, vapour pressure, and surface tension. High viscosity promotes wave damping, decreases the number of active cavitation zones, and reduces process efficiency. Therefore, it is preferable to use low-viscosity liquids; if the viscosity is high, the amplitude should be increased [142]. Solvents with lower vapour pressure are preferred because high vapour pressure reduces the energy released during bubble collapse. Bubble formation is more difficult in liquids with high surface tension, yet such liquids, including water, exhibit higher cavitation activity [140]. As the temperature of the liquid medium rises, both viscosity and surface tension decrease, while vapour pressure increases. As a result, higher temperatures generally reduce cavitation intensity. It can also degrade thermosensitive components. On the other hand, an increase in temperature can favour the rate of chemical and biochemical reactions. Therefore, the recommended temperature level must be selected for the specific system. Due to the rapid temperature rise during ultrasonication, cooling should be employed to maintain the desired temperature [143]. Gases dissolved in a liquid medium can positively influence cavitation by serving as nuclei for cavitation bubble formation.

Ultrasound pre-treatment offers significant benefits, but its effectiveness depends on understanding the factors that influence it. Selecting optimal parameters and maintaining consistent experimental conditions are essential. It is essential to consider material properties, making parameter optimisation critical for each application.

### 3.7. Infrared Radiation

Traditional blanching in water or steam is an important pre-treatment before drying. However, it has its drawbacks, including the leaching of soluble components and increased water content. Therefore, researchers are exploring alternative methods to achieve a similar effect, using infrared radiation (IR). The primary goal of blanching is to inactivate enzymes responsible for undesirable changes in colour, flavour, and texture. IR spans the electromagnetic spectrum between visible light and microwaves, with wavelengths ranging from 0.78 to 1000 μm. IR is divided into three categories based on wavelength: near-infrared (NIR, 0.78–1.4 μm), mid-infrared (MIR, 1.4–3.0 μm), and far-infrared (FIR, 3.0–1000 μm). When infrared radiation strikes a body that absorbs it, it is converted into heat. No resistance to heat transfer accompanies this process. Therefore, the efficiency of this type of heating is high. MIR and FIR are primarily used for surface heating of high-water content food products [79], as water exhibits strong absorption bands in these regions (2–14 µm). Sources of infrared radiation used in technological applications include electric heating elements (tungsten, ceramic, quartz), gas heaters, catalytic heaters, and the sun. The emitters are typically placed above or below the product. Catalytic CIR emitters are characterised by high efficiency, reaching 80–90%, while consuming nearly half the energy of EIR emitters. In this type of emitter, a gas (natural gas or propane) is oxidised on the surface of a catalyst (platinum or palladium), and the resulting heat from an exothermic chemical reaction is emitted as IR [11,144]. Depending on the emitter type, they heat from 300 to 700 °C for ceramic emitters to up to 2000 °C for quartz emitters. The higher the emitter temperature, the shorter the wavelengths emitted. Shorter wavelengths penetrate deeper beneath the heated surface, to a depth of 1–3 mm, whereas longer wavelengths penetrate to several dozen micrometres. Infrared radiation is strongly absorbed by water, making it ideal for heating food products, which typically contain a lot of water. The ability to absorb radiation is influenced by water, protein, and fat content. The absorption ability also depends on the material’s structure—in dense products, penetration is lower, and in porous materials, penetration is greater. The colour and optical properties of the surface also determine absorption: dark materials absorb more radiation, while light and shiny materials absorb less. Therefore, two different materials heated in the same field can reach different temperatures. The heating effect depends on the dominant wavelength emitted by their power emitters, the emitted power density, and the distance between the emitter and the heated surface. Doubling this distance results in a fourfold increase in the incident radiation intensity [145].

The primary purpose of blanching is to deactivate enzymes that break down food components, which helps preserve the colour, flavour, texture, and nutrient content during the drying process. One of the key enzymes targeted for deactivation is polyphenol oxidase (PPO), commonly found in various fruits and vegetables, as it is responsible for enzymatic browning. This process leads to the oxidation of phenolic compounds into quinones in raw materials like apples and bananas [146]. Another enzyme important in browning is peroxidase POD, which reacts with hydrogen peroxide to form phenoxy radicals. As a result of this reaction, coloured compounds such as chlorophyll can be oxidised, leading to unfavourable colour changes. The presence of PPO and POD enzymes is commonly used as a measure of blanching effectiveness, including IRB. However, the effect is usually superficial and more difficult to achieve throughout the sample [147]. IRB can be successfully used to increase or reduce the microbiological purity of raw materials to be dried. Infrared radiation energy is absorbed by water within microbial cells, raising temperatures and denaturing proteins, disrupting microbial cellular structures. At the same time, the enzymes within cells are inactivated [148]. Using NIR blanching, it is also possible to inactivate resistant microbial spores, such as B. subtilis and Aspergillus Niger [12]. For example, complete inactivation and degermination of B. subtilis spores were achieved after IR irradiation at 14–15 GW/m^2^ [149].

IR blanching is more effective than hot water blanching. Direct energy transfer to the material surface ensures high thermal efficiency, minimal changes in product quality, and reduced processing time and energy costs [15]. The use of IRB helps preserve the colour, structure, and overall sensory quality of dried fruits and vegetables. It can also shorten the drying time. Wu et al. [147] found that the drying rate of IR-blanched and convection-dried carrots was higher, and the drying time was shorter. Vitamin C retention was also higher, and the dried samples exhibited better rehydration capacity, less colour change, and less shrinkage than heat-blanched samples dried under the same hot-air conditions. However, drying the potato after IRB treatment improved colour, vitamin C retention, and enzymatic activity, but the drying rate was slower [150]. In this case, IRB likely intensified starch gelatinisation on the potato surface, leading to the formation of a crust that hindered water evaporation. Numerous studies have shown that vitamin C retention increases due to the inactivation of certain enzymes, including ascorbate oxidase. The literature reports numerous benefits of infrared blanching (IRB) compared with hot-water or steam blanching, including improved retention of phenolic compounds, carotenoids, and other bioactive compounds. However, there has been limited focus on optimising blanching conditions.

### 3.8. UV-C Light and Pulse Light

The ultraviolet (UV) spectrum encompasses wavelengths from 200 to 400 nm and can be categorised into three types: UVA, UVB and UVC [151]. Short-wave ultraviolet light (UV-C) treatment is mainly used for sanitising and retarding microbial growth on food surfaces, especially fresh-cut fruits and vegetables. It is based on non-ionising radiation in the electromagnetic spectrum ranging from 200 to 280 nm [152]. The use of UV-C treatment before the drying process is quite uncommon. Ramanathan et al. [153] analysed the synergistic effects of UV-C treatment during microwave drying of *Muntingia calabura* leaves. The 5–10 min application of UC-C, in conjunction with microwave drying at 200 W, resulted in higher moisture diffusivity and reduced drying time and energy consumption. Longer UV-C treatment at high microwave power led to tissue damage and pigment loss.

High-intensity pulsed ultraviolet light (HIPL) is an effective non-thermal method for sanitising fruits. UV systems can emit energy at a specific wavelength, while pulsed UV light systems deliver intense radiation across a broad spectrum, including UVA, UVB, UVC, and visible light. This technology delivers short pulses of energy that are absorbed by the fruit’s tissue, converting into internal energy and causing localised moisture evaporation. During evaporation, a rapid increase in volume occurs, leading to cell rupture [151].

Pulsed light (PL) is also referred to by several terms, including pulsed ultraviolet light (PUV), intense light pulse (ILP), high-intensity broad-spectrum pulsed light (BSPL), high-intensity pulsed UV light (HIPL), and pulsed white light (PWL). Light pulses are produced by stimulating inert gases, such as xenon, in flashlamps and by collisions of gas molecules triggered by an electrical pulse [154]. In the treatment of mangoes, a series of 10 to 50 ultraviolet light pulses was applied. The spectral composition of the light comprised 30% UV radiation, 30% infrared radiation, and 40% visible light. The pre-treatment did reduce drying time. However, the dried samples treated with fluences in the range 3.6 to 10.8 J/cm^2^ contained 10–40% higher levels of vitamin C and carotenoids than the untreated mango. Pulsed UV treatment significantly degraded vitamin B6, which is highly light-sensitive [151].

Using intensive pulsed light (IPL) with 25 pulses at 400 J before drying shiitake mushrooms decreased enzyme activity due to structural changes in the enzymes. The pre-treatment method significantly reduced the brown index and the degree of browning, while enhancing the colour and antioxidant activity of dried mushrooms [155]. Pre-treatment with pulsed light (PL; 1000 mJ/cm^2^, 15 flashes) reduced colour deterioration by inhibiting enzymes during vacuum freeze-drying of Chinese bayberry. Additionally, combining PL with freeze–thaw cycles improved the retention of bioactive compounds [156]. Pulsed light treatment of food is mainly utilised for microbial decontamination and may also help reduce its moisture content. Additionally, this method shows promise for preserving bioactive compounds in dried foods. When combined with other processing techniques, pulsed light treatment can offer additional advantages, such as decreased drying time.

### 3.9. Cold Plasma

Cold plasma is an emerging non-thermal pretreatment technology before drying. This method is widely used in food preservation and disinfection due to its antimicrobial properties. Plasma can be considered a state of matter distinct from liquids, solids, and gases. Plasma can be defined as an ionised gas that contains a mixture of positive and negative ions, free radicals, photons, neutral particles, reactive oxygen and nitrogen species, reactive nitrogen species molecules, and atoms in different states. The application of electrical energy to gases such as argon, helium, air, oxygen, and various gas mixtures results in ionisation and the formation of plasma [65,157,158].

Depending on the electron temperature, plasma can be classified as thermal or non-thermal. Cold plasma is considered non-thermal as it operates at temperatures between 30 and 50 °C, making it suitable for processing heat-sensitive materials. There are several methods for generating cold plasma, each relying on different plasma discharge mechanisms. One such method is the dielectric barrier discharge technique, which generates plasma via an electrical discharge betweens two electrodes separated by an insulating barrier. As gas travels between the electrodes, it ionises, forming plasma. A high-voltage alternating-current or direct-current discharge is used in the process. The corona discharge technique creates plasma by changing the electric field strength at atmospheric pressure. In the plasma jet discharge method, carrier gas ionisation occurs between the electrodes in the jet system, driven by radio-frequency fields. Another method for creating plasma uses a magnetron, which generates microwave plasma through a high-frequency electromagnetic field [159].

The application of cold plasma may cause many physical and chemical reactions which significantly alter food properties. Numerous studies have demonstrated that pre-treatment with cold plasma before drying enhances both the efficiency of the dehydration process and the quality of the final products [157]. Samples treated with cold plasma dried faster due to increased effective moisture diffusivity. Cold plasma application reduced dehydration time of carrot slices by 9.68–12.90% in vacuum drying and 22.22–37.04% in tray drying. The analysis of the microstructure of dried carrots showed that the formation of micropores and surface loosening were key factors that enhanced moisture transport during drying. Additionally, cold plasma application reduced degradation of carotenoids and phenol content and noticeably enhanced antioxidant capacity compared with untreated dried samples. However, the extent of these changes was influenced by the timing of cold plasma treatment and the drying method [160]. Similar beneficial results, including shorter drying times and improved retention of many bioactive compounds, were observed for dried chilli pepper [161], jujube [162], wolfberry [163], and white grapes [164] treated with cold plasma pre-treatment. The enhanced drying efficiency was associated with alterations in the internal structure, specifically in the penetration depth of plasma reactive species. This adjustment can help prevent case hardening and reduce the falling rate period during the drying process [165]. The disruption of tissue aggregates could enhance bioactive compounds and their activities by facilitating the release of cellular constituents [65].

In terms of additional physical properties, applying cold plasma treatment to dried materials resulted in either no effect or a significant enhancement in colour quality. Bao et al. observed undesirable colour changes in dried samples with prolonged cold plasma treatment [162]. The application of cold plasma can affect the enzyme activity. Mainly, the process reduced polyphenol oxidase and peroxidase activities in the dried materials. However, the effect of cold plasma pre-treatment on the reduction or intensification of browning was related to the type of product or tissue [157]. Manafi et al. [166] reported that cold plasma also reduced shrinkage and improved the rehydration ratio of air-dried red beet. However, pre-treatment parameters, such as plasma application times, infrared power, and pulse ratios, modified the magnitude of the observed changes in material properties.

The use of cold plasma offers numerous advantages, particularly in enhancing the quality and safety of dried products while reducing drying time, thereby lowering energy consumption. This technology can be easily integrated into a laboratory’s technological line. However, the currently available equipment is still quite expensive. Additionally, managing the process—which is multivariate and nonlinear—presents challenges. Monitoring the process parameters and conditions to achieve the desired characteristics of dried products is also complicated. As a result, the scaling up of this technology within the drying industry is still in its early stages [157]. Miletić et al. [167] reported that the Technology Readiness Levels (TRLs) of cold plasma technology are currently assessed at levels 4–5. This classification suggests that the technology is still in the early stages of development, having been validated in laboratory and relevant environments. The progression to higher TRL classifications is currently impeded by the complexities of designing equipment that ensures uniform surface treatment of sliced products. The methodologies also necessitate the optimisation of parameters such as power, exposure duration, and gas composition, tailored to various food types for industrial applications [165]. The utilisation of cold plasma technology is characterised by its low electrical power consumption and the concomitant reduction in drying time, resulting in significant energy savings. These attributes contribute to the sustainability of this technology [167].

### 3.10. Coating and Dipping in the Solutions

Edible coatings are thin layers, typically less than 0.3 mm thick, that cover the surface of food [168]. Coating can be a pre-treatment applied before drying to enhance material quality by reducing colour and aroma loss, improving the retention of bioactive compounds, and extending shelf life. The resulting coating provides additional resistance to water transport but also acts as a barrier to oxygen in the drying air. Edible biopolymer coatings are primarily prepared from aqueous solutions containing carboxymethyl cellulose, starch, and chitosan. Various methods, such as immersion, rolling, and spraying, can be used to apply these coatings to food [135].

Coating can influence moisture transfer during drying, leading to more uniform dehydration. This process may enhance the preservation of texture and tissue integrity, although it does not always reduce drying time [169,170]. A coating of starch and calcium gluconate salt was applied to papaya before air-drying. The coating acted as a moisture barrier, which affected the drying process. The drying rate decreased as the coating addition increased from 0 to 3% [171]. Li et al. [169] used the sodium carboxymethyl cellulose (CMC) solution at a concentration of 0–2.5% to form a layer on the *Zanthoxylum schinifolium* fruits. The drying time of coated samples containing 1% and 1.5% CMC was similar to that of the pre-treated control material. Furthermore, increasing the CMC concentration to 2.5% extended the drying time by 16.7%. The observed behaviour can be explained by a barrier effect and the hydrophilic properties of carboxymethyl cellulose (CMC). Conversely, applying a sodium alginate solution to purple-flesh potatoes at concentrations ranging from 0.2% to 0.8% significantly reduced drying time by approximately 8 to 10 min compared with uncoated samples. The presence of alginate and sodium ions influenced the dielectric properties, resulting in improved conductivity and enhanced thermal heat transfer during microwave drying [172].

The presence of a coating layer prevented rapid surface drying of cherries and the formation of cracks and non-uniform moisture distribution in the dried tissue. Coatings with sodium carboxymethyl cellulose and sodium alginate also successfully preserved the colour, taste, and nutritional qualities of cherries [170]. However, hardness increased, while shrinkage in dried papayas decreased as coating concentration increased. The application of a coating with starch and calcium gluconate salt reduced thermal losses of bioactive compounds in dried fruits [171]. Coatings containing native and modified maize and cassava starch were successfully used to retain carotenoids during air-drying [173]. The application of a pre-treatment with a CMC solution demonstrated significant efficacy in preserving the integrity of plant essential oils during drying. Notably, fruits treated with a 2.5% CMC coating exhibited a remarkable 100% retention rate, underscoring the potential of this method to enhance the stability of volatile compounds [169]. Microwave-dried potatoes coated with sodium alginate were less porous, thereby reducing the rehydration ratio and enhancing the retention of purple colour compared to uncoated dried samples [172]. The quality of dried products that underwent coating pre-treatment is influenced by various factors, including the type of coating used, its thickness and concentration, the characteristics of the raw materials, as well as the methods and conditions employed during the drying process.

The selection and optimisation of the coating formulation, as well as the control of coating migration, can also be challenging. The main limitations of the application of edible coatings as a pre-treatment technology can be related to thick coating layers, which may reduce the drying rate. Another challenge with the broader use of this technology is the significant initial capital investment and the difficulty of scaling [174]. The laboratory scale is well established, but scaling it up to industrial levels presents significant economic and technical challenges.

Another pre-treatment used for food immersion in solutions is the application of calcium chloride. When plant tissue is dipped in a calcium ion (Ca^2+^) solution, the solution influences the tissue’s structure and texture. This pre-treatment increased cell wall thickness and enhanced the tissue’s resistance to deformation during the drying of red bell peppers. As a result, the damage to the structure was minimised by this pre-treatment [175]. The increase in tissue strength was attributed to the interaction between calcium and pectin acid in the middle lamella, forming CA-pectate [176,177]. Immersing apples and potatoes in a calcium chloride solution at 20 °C before microwave-assisted drying affected the strength of plant tissue cell walls and increased the hardness of rehydrated materials compared to samples without pre-treatment. However, calcium infusion did not significantly affect the drying rates of apples. Water diffusivity can be influenced by various factors, such as tissue type, drying temperature, and pre-treatment temperature [177]. Also, calcium concentration and pre-treatment duration can affect the drying process and the material’s properties. The papaya samples were treated with calcium chloride at concentrations ranging from 0.5 to 2.5%, then osmotic-dehydrated and convection-dried at 70 °C. Calcium-treated dried papaya had lower water content and shrinkage than untreated dried samples. However, adding 2.5% calcium caused undesirable brittleness in dried papaya. Replacing calcium chloride with calcium lactate (CA-L) improved the sensory characteristics of the dried product [176].

The application of ethanol pre-treatment mainly intensified the drying process. Generally, alcohol at concentrations above 70% possesses disinfectant properties due to the denaturation of enzymes and microbial proteins caused by dehydration. Ethanol can dissolve cell wall components, leading to changes in membrane structure and permeability [178]. The increase in drying speed observed in samples treated with ethanol can be attributed to ethanol’s interaction with water, creating a mixture with higher vapour pressure [179]. Soaking eggplant slices in a 15% ethanol solution for 5 min significantly reduced their drying time by more than 50%. Furthermore, the treated samples exhibited enhanced colouration and rehydration ratios, compared to untreated materials [180]. A similar effect was observed in infrared hot air-dried scallion when soaked in 75% ethanol for 10 min. The ethanol-treated samples showed improved rehydration, odour retention, vitamin C preservation, antibacterial properties, and considerably shorter drying times [178]. The enhanced drying rate and decreased drying time were observed in many studies with applied ethanol pre-treatment compared to control samples; for mango peels [143], beetroot [144], and pumpkin [181], drying time was reduced by 28, 30, and 48%, respectively. The pre-treated pumpkin samples also showed near-100% preservation of carotenoids [181]. Better retention of colour, higher content of many bioactive compounds, and greater antioxidant capacity were observed in ethanol-pre-treated and dried apples [182], mango [183], yacon [184], and melon [185]. Reducing the duration of heat exposure during the drying process is associated with reduced material degradation and significant energy savings. For instance, applying this enhanced drying method to rose petals resulted in energy consumption that was 64% lower than for samples dried without treatment [186]. A key factor limiting the implementation of this treatment is the need to optimise it for process conditions. Given alcohol’s flammability, safety concerns must be addressed regarding its use.

### 3.11. Enzymatic Treatment

The application of enzymatic treatment before drying is uncommon, and the literature reports few documented instances. Nevertheless, existing research highlights substantial potential for implementing this technique.

The use of enzymes for dewaxing serves as a gentle drying pre-treatment method that conserves energy and minimises chemical pollution. Also, during the treatment, no high temperatures are observed. Their application after harvesting enhances the permeability of the outer membranes and facilitates drying [187]. Sun et al. analysed the use of a mixture of enzymes, including cutinase, cellulase, and pectinase, to degrade cell wall components in plant tissues. This enzymatic treatment enhanced the permeability of cherry tomato peels, significantly accelerating the drying process. Additionally, the lycopene and phenol contents in tomato increased due to enzymatic treatment [188]. Application of different enzymes (chitinase, pectinase, cellulase, cutinase) in the pre-treatment at 50 °C of goji berries resulted in shortening vacuum-drying times by 18–57.29%, depending on enzyme types. The reduction in the density of the fruit’s waxy layer and the formation of micropores facilitated the moisture transfer during drying. This pre-treatment also contributed to retention colour, polyphenol, and flavonoid levels, as well as improved rehydration rates compared to untreated wolf berries [187]. The application of pectinolytic enzymes to blueberries before freeze-drying has been shown to enhance their physical integrity. Blueberries subjected to freeze-drying without any pre-treatment exhibited significant internal structural damage, characterised by the formation of large voids. Conversely, the blueberries treated with enzymes displayed a microstructure characterised by numerous small pores. The enzymatic treatment slightly reduced the polyphenol content and halved the drying time compared with the dried, untreated blueberries [36]. Application of pectinolytic and cellulolytic enzyme preparations for different durations (20–60 min) before freeze-drying showed that the pre-treatment duration affected both the drying time and the quality attributes of blueberries. Pectinolytic enzymes significantly enhanced the drying rate compared to cellulolytic enzymes. The 30 min pre-treatment reduced the drying time by 3-fold. Enzymatic pre-treatment with pectinases enhanced the physical characteristics of the dried fruit compared with untreated dried berries [189]. However, treatment with pectin lyase and polygalacturonase pumpkin apple did not influence the drying times [190].

Numerous factors influence the effectiveness of enzymatic treatments in enhancing plant tissue membrane permeability, including the type of enzyme, concentration, exposure duration, and tissue structure. This pre-treatment has been primarily applied to waxy fruits, as conventional methods were often prolonged and less effective. Understanding these variables is essential for optimising enzyme use in the drying process.

### 3.12. Carbon Dioxide Treatment

Carbon dioxide treatment can be employed via carbonic maceration, in which the plant material is subjected to a carbon-rich atmosphere. During this process, plant tissue undergoes anaerobic metabolism. Due to these conditions, modifications in cell membrane structure, inactivation of many enzymes, and cellular damage can occur. It also leads to more acidic conditions, in which the retention of many bioactive nutrients can be maintained. Also, some studies have shown that applying carbon maceration as a pre-treatment method decreased drying time [30].

On the positive side, CM effectively enhances mass transfer during drying, significantly reducing drying times. For instance, studies show that carbonic maceration at 1–5 MPa for longan (*Dimocarpus longan* Lour) decreased drying time by 72.62–76.79%. Similar results were obtained during drying of raisins [131], sweet potato [132], and tomato [191]. The reduction in drying time and the increase in mass transfer during vacuum microwave dehydration were also observed for CM-treated ginger. The pre-treatment created a porous structure, reducing the drying time of ginger by 42.27% [192]. This reduction is largely attributed to increased cell membrane permeability, which improves the extraction of beneficial compounds. As a result, the total phenol content, vitamin C, flavonoids, anthocyanins, and β-carotene levels in dried materials were significantly higher than in untreated samples. The results showed a notable increase in antioxidant activity after pre-treatment. Multiple studies have confirmed that carbonic maceration (CM) pre-treatment significantly improves the physicochemical and biochemical properties of dried materials [193,194,195].

While the CM process facilitates the release of beneficial compounds, it also degrades cellular structures, particularly vacuoles. This disruption, while beneficial for nutrient extraction, can adversely affect the texture and structural integrity of the final product. For example, dried tomatoes subjected to CM show reduced firmness, skin strength, and elasticity, which can deter consumer acceptance. Due to damage to the cell wall integrity of plant tissue, mechanical strength was reduced, and the structure became more tender after drying [191]. Additionally, CM pre-treatment is considered environmentally friendly as it produces no undesirable residues from chemical reagents. However, the extended maceration process (12–72 h) may reduce processing efficiency and increase production costs compared with conventional pre-treatments. Longer processing times may hinder the scalability of this pre-treatment method [30]. The effectiveness of carbonic maceration (CM) relies on the optimisation of several parameters, including temperature, pressure, and time. This process may require complex monitoring systems. Additionally, the impact of carbonic maceration varies depending on the structure and texture of different foods. It is important to note that this technology requires specialised equipment, which can increase initial investment costs. Scaling up the process can also be challenging due to nonuniform gas distribution [191,194].

An alternative method for using carbon dioxide in food processing before drying is CO_2_ laser beam technology. This is an emerging technique that operates in the mid-infrared spectral range, utilising a gas mixture of carbon dioxide, nitrogen, and helium. This technique involves electron collisions that excite nitrogen molecules, which then transfer energy to carbon dioxide (CO_2_) molecules, with nitrogen serving as an energy intermediary [196,197]. Laser technology enables the creation of accurate micro-perforations at specific points on various surfaces and in various patterns, with diameters ranging from 50 to 300 μm. This technique can serve as an alternative to needle puncture. Applying a carbon dioxide laser before drying accelerates mass diffusion and increases the drying rate [198]. This technology has significant potential; however, it is still in development and needs further refinement for industrial applications. Munzenmayer et al. [199] conducted a study in which they used a carbon dioxide laser to create microperforations in blueberry skin before freeze-drying. The results showed that the primary drying time was reduced by 23.5% for blueberries with nine micro-perforations compared with those left untreated and whole. Also, the application of CO_2_ laser technology reduced the primary freeze-drying time of strawberries by 20% when five micro-perforations were made. The strawberries with micro-penetration showed better rehydration compared to freeze-dried samples without pre-treatment [198]. A similar effect was observed for laser-treated strawberries microwave-freeze-dried. The reduction in drying time by 23.08% and the shrinkage rate by 3.95%, as well as an increase in the anthocyanin retention rate by 20.02%, were observed with the application of carbon laser pre-treatment [200].

This laser technology holds great potential; however, it is still under development and requires further refinement to be adapted for industrial use.

## 4. Comparative Analysis of Pre-Treatment Methods Before Drying

A thorough review of the literature on pre-treatment processes before drying reveals a significant lack of justification for the selection of specific methods. This gap highlights the need for a more rigorous evaluation of the criteria guiding the choice of pre-treatment techniques to optimise drying efficiency and product quality.

**Figure 5:**
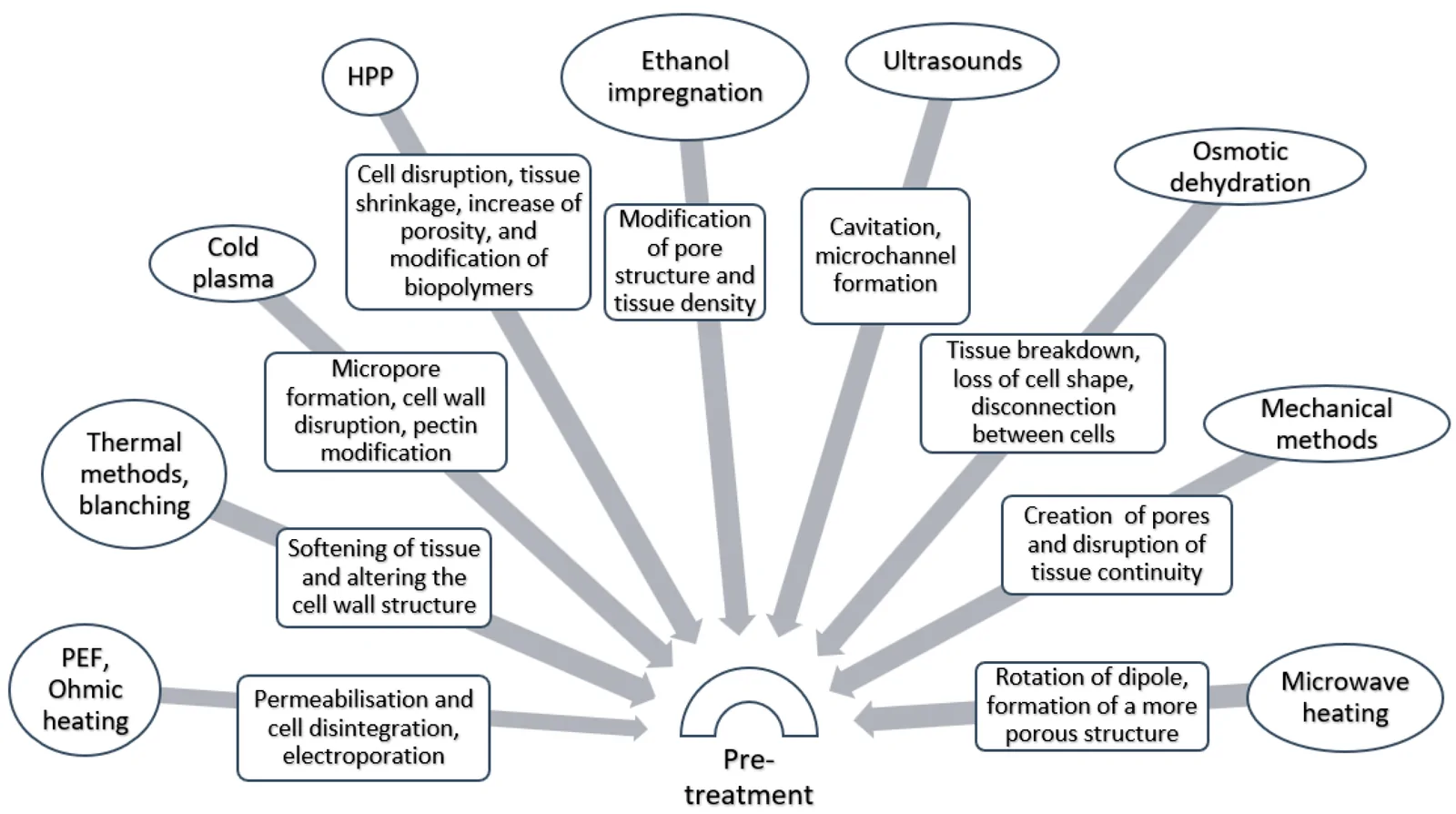
Structural changes associated with the application of pre-drying treatment.

This review underscores the mechanisms underlying each processing method, thereby facilitating the design of effective processes. Figure 5 illustrates the principal structural alterations observed following various pre-treatment methods. These modifications may significantly influence the dynamics of the drying process, potentially leading to variations in drying time, which in turn affects the overall quality of the materials. Numerous properties of the materials under consideration can be correlated with the pre-treatment methods employed, underscoring the importance of these techniques in optimising material characteristics during subsequent drying. Drawing on our extensive experience, we present the following guidelines: There is a prevailing trend toward minimising production costs, which does not favour adopting modern processing methods, which are often costly. Moreover, the consideration of unit operating costs is critical, as the raw materials used for drying are relatively inexpensive and readily accessible. Thus, the pricing of dried products should remain reasonable. However, a notable segment of premium products with high nutritional value and quality necessitates research into optimal pre-treatment methods. Researchers and technologists must wisely select these methods, guided by an understanding of the transformations they may cause. The main objective is to reduce drying time significantly enough to justify the chosen processing method. Techniques that may damage the cell membrane should be employed to achieve shorter drying times throughout the material’s volume.

Table 1 provides estimated, indicative values resulting from the mechanisms of action and the depth to which the desired changes can be achieved. These are usually in the range of several to a dozen or so millimetres. Distinguishing methods include ohmic heating and high pressure. In these cases, the desired effect can be achieved throughout the entire volume, regardless of the material’s size. Microwave blanching is also worth considering. Although the depth of microwave exposure is limited to a few centimetres, this value usually does not exceed the size of the plant materials. Pulsed electric field treatment also seems to be a good choice; however, in this case, electro-pervaporation releases cellular enzymes, which can cause unfavourable colour changes. It is not justified to use methods that mainly affect the surface when the goal is to reduce drying time.

For raw materials with waxy skins, methods that primarily reduce skin resistance should be used. In this case, steam blanching, enzymatic treatment, or carbon dioxide treatment integrated with an NIR laser (Table 1), which creates micropores in the skin, seems to be good choices. In addition to reducing drying time, another often overlooked aspect of pre-treatment is the microbiological quality of dried materials, particularly herbs and spices. Therefore, improving the microbiological purity of the raw material, and thus the dried material, is also a very important aspect of method selection. To achieve this goal, cold plasma or steam blanching appears to be an effective solution, especially since these methods do not entail excessive investment or operating costs.

One critical consideration in selecting a pre-treatment method is preserving high nutritional value. Nonetheless, it appears that selecting an appropriate drying method plays a more pivotal role in achieving this objective.

Optimising pre-treatment parameters is essential, regardless of the main goal or processing method chosen. Ultrasound, cold plasma, and ohmic heating are particularly demanding in terms of parameters and technical conditions. These parameters are discussed in detail in Section 3.4, Section 3.6 and Section 3.9. It is important to note that achieving the intended goal requires optimising these parameters for the specific material. Due to this particular sensitivity to various parameters, scaling up production may also be difficult for these processes. The technology readiness level of the process is relatively low, falling between TRL 4 and 5. When selecting processing conditions, it is crucial to establish a simple methodology for monitoring treatment-induced changes to assess treatment effectiveness (Table 1). In the PEF method, measuring the electrical conductivity of the tissue after processing (which increases with the amount of cell sap released) enables easy assessment of the extent of tissue changes and quick selection of appropriate conditions. Specific energy consumption (SEC) in drying is affected by various factors, including the raw material properties, processing parameters, drying methods, and conditions. This complexity underscores the need for a nuanced understanding of the drying process.

Selecting the most suitable pre-treatment method for a specific process necessitates careful consideration of the inherent characteristics of the plant tissue involved. A proposed classification framework considers several parameters, including the structure’s dimensions, hardness or softness, the presence of a protective skin layer, and the presence of a leafy morphology (Table 2). Based on the above literature review and our own experience, the authors recommend groups of methods most appropriate for tissues with specific characteristics. Steam blanching is considered the most universally applicable, simple, and cost-effective method for food processing. In contrast, pulse electric field (PEF) technology is also broadly applicable but incurs substantially higher implementation costs. 

## 5. Conclusions and Future Directions

The distinction between traditional and unconventional pre-drying methods in this study is arbitrary; what is considered modern today may soon be adopted widely. Additionally, modifications to traditional methods can introduce innovative features. A variety of material pre-treatment techniques before drying have been discussed, with multiple factors influencing their application in industrial practice. When selecting an appropriate treatment for a technological process, it is essential to define its objectives clearly. In the context of drying, the primary aim is to accelerate the process while preserving product quality in both physical and nutritional aspects. When assessing pre-treatment methods, it is crucial to identify key barriers to heat and mass transfer, such as raw material thickness, particle size, and the presence of cellular membranes or outer skins. Additionally, one must consider the inherent variability of biological materials arising from diverse varieties or species, as well as temporal changes influenced by factors such as maturation. A comprehensive understanding of the underlying mechanisms and relevant factors affecting treatment efficacy is vital for selecting the optimal method. Small modifications in treatment conditions or parameters can significantly influence drying outcomes. A suboptimal result should prompt a reassessment of process conditions rather than a complete abandonment of the method. Thus, optimising pre-treatment conditions for each specific raw material is imperative, as effects can vary by material type. Applying pre-treatment methodologies indiscriminately from the existing literature is impractical. From an economic perspective, minimising drying time is critical when selecting a pre-treatment method. Additionally, the substantial costs of equipment and the complexities of scaling laboratory experiments to industrial applications must be addressed. Controlling pre-treatment process parameters is vital because they directly affect the final quality of dried food products. The choice of drying method and its specific parameters should be grounded in a thorough understanding of product quality, economic evaluations, and equipment availability. In an industrial context, factors such as production scale and operational efficiency are essential. Given the multifaceted nature of these aspects, no singular optimal pre-treatment method exists; the selected approach must be carefully tailored to the material’s behaviour throughout the drying process.

## Figures and Tables

**Table 1:** Effect of different pre-treatment methods on effectiveness, technology readiness level (TRL), and specific energy consumption (SEC) in drying.

Type of Pre-Treatment	Depth of Changes Caused by Treatment *	Factors Influencing the Depth of Changes	Possibilities of Monitoring the Effectiveness of Pretreatment	RTL [167,201]	SEC [167,201,202]
Water blanching	2–10 mm	Time, temperature, thermal properties of the material, dimensions and shape of the material	Evaluation of the activity of specific enzymes: PPO (polyphenol oxidase), POD (peroxidase), PME (pectin methylesterase), and LOX (lipoxygenase)	9	7–14 kWh 7–114 kWh/kg
Steam blanching	2–10 mm	Time, temperature, thermal properties of the material, dimensions and shape of the material	Similar to water blanching.	9	6–39 kWh 29–42 kWh/kg
Soaking in citric acid solutions	1–5 mm	Tissue structure, processing time, solution concentration, temperature	Measurement of pH or citric acid concentration using analytical methods for the subsequent layers of the product	5–7	0.7–2.6 MJ/kg Lower SEC and drying time
Sulfurization (SO_2_/sulphites)	2–8 mm	Tissue structure, processing time, solution concentration, and temperature	Determining the sulphite content and enzyme activity in the subsequent layers	6–8	Slightly lower drying time
Osmotic dehydration	Unlimited	Tissue structure, processing time, solution concentration, temperature, type of osmotic agent, circulation	Monitoring of losses of water and solute gain, analysis of texture changes	8–9	4–6 kWh 155–1200 kW/kg
Microwave blanching	Throughout the volume, the layer is no more than a few centimetres thick.	Water content, material structure, thermal properties of the material, and microwave frequency	Similar to water blanching	6–8	Lower SEC and drying time
High-pressure Processing	Unlimited	The entire cross-section of the product, regardless of its shape, size, or structure, is to be considered.	Activity of selected enzymes, as for blanching.	8–9	Lower SEC and drying time
Ohmic heating	Unlimited	Electrical conductivity of foods, intensity and frequency of electric field, heat capacity, and the electrode material	Activity of selected enzymes, as for blanching.	5–6	Lower SEC and drying time
Pulsed electric fields	Up to a few centimetres	Chamber geometry, electrical conductivity of the raw material, electric field strength, and material homogeneity	Assessment of tissue conductivity and texture.	6–8	9–463 kWh/kg
Ultrasounds	From a few millimetres to a few centimetres,	Frequency, amplitude, ultrasonic power, system geometry, viscosity, material structure	Microstructure image, e.g., SEM, showing cell damage, texture assessment.	6–7	1–7 kWh 6–240 kWh/kg
Infrared Radiation	Surface effect	Wavelength, radiation intensity, distance from the irradiated surface, surface type and colour, chemical composition	As for blanching	8–9	Lower SEC and drying time
UV-C Light and Pulse Light	Surface effect	Water content, structure, presence of coloured substances	Microstructure and texture assessment.	4–6	Limited impact on the reduction in time and energy
Cold Plasma	Surface effect	Short duration of reactive oxygen and nitrogen species—RONS	Degree of surface microorganism reduction, enzyme activity, as for blanching.	4–5	1–9 kWh 2–33,347 kWh/kg
Coating	Not applicable	Not applicable	Microscopic image of the coating.	4–6	Limited effect
Dipping in ethanol	5–15 mm	Treatment time, solution concentration, temperature, tissue structure	Chemical analysis of individual layers.	4–6	4.5–7.0 MJ/kg Lower SEC and drying time
Dipping in Ca^2+^ solution	1–5 mm	Treatment time, solution concentration, temperature, tissue structure	Chemical analysis of individual layers.	3–4	Limited effect
Enzymatic treatment	The impact on the skin, which serves as the primary barrier to mass transport,	Enzymatic activity, treatment time, treatment conditions	Analysis of mechanical properties.	4–6	Lower SEC and drying time
Carbon dioxide treatment and laser-assisted	Surface effect	The laser enhances penetration depth by several millimetres, reaching a maximum of 5 mm for NIR wavelengths.	Measurement of the pH levels in individual layers.	4–6	Limited effect

* Values estimated based on own measurements or analysis of phenomena resulting from mechanisms of unsteady heat, mass transfer, and radiation absorption.

**Table 2:** Recommendations for selecting the type of pre-treatment before drying based on material characteristics, excluding specific drying methods.

Tissue Characteristics	Recommended Processing	Main Potential Benefits	Disadvantages	Aspect CAPEX/OPEX	Environmental Footprint/Net Sustainability
Large size, over 2 cm	Grinding to particles less than 1 cm	Reduced drying time, ease of dosing	Possible leakage	Low capital expenditure, low operating costs	Higher energy composition, a waste generation
Hard structure (vegetables, some fruits, e.g., carrots, potatoes, apricots)	Steam blanching	Reduced drying time while maintaining nutrients	Large temperature difference between the surface and the centre	Low capital expenditure, low operating costs	Energy-efficient, food waste valorisation, problem of overheating
	Microwave blanching	Reduced drying time while maintaining nutrients	Possibility of local material overheating, need to select processing parameters for a given material	High capital expenditure, short pre-treatment time, low operating costs	Minimised adverse footprints, lower energy use
	Infrared blanching	Reduced drying time while maintaining nutrients	Possibility of surface overheating,	Low capital expenditure, low operating costs	Water and waste reduction, lower energy consumption
	High-Pressure Processing	Reduced drying time while maintaining nutrients	Necessity of liquid phase mediation, possibility of protein chemical transformations, long processing time	High capital and operating costs	Green and sustainable, with a lower carbon footprint than thermal methods
	PEF	Reduced drying time while maintaining nutrients	Necessity of individual parameter selection	High capital and operating costs	Low-energy, environmentally friendly, green technology, more efficient, less waste
	Ultrasound	Reduced drying time while maintaining nutrients	Necessity of liquid phase mediation, effect very dependent on technical processing conditions, need for cooling during processing, potential for free radical formation	Moderate capital and operating costs	Green technology, reduced energy consumption,
	Ohmic heating	Reduced drying time while maintaining nutrients, reducing mould and yeast growth	Difficulty in temperature control, need for optimisation of processing parameters, need for immersion in the solution, heating intensity dependent on electrical conductivity, high safety requirements	High capital expenditure, average operating costs	Lower energy consumption, time savings
Soft structure (soft fruits, e.g., strawberries, blackberries)	Ultrasound	Reduced drying time, uniform thermal degradation of cell membranes, reduced colour changes, enzyme deactivation, short processing time	The necessity of liquid phase mediation, excessive softening of the structure, may lead to the formation of free radicals, multiple parameters affecting the effect	Moderate capital and operating costs	Analogously to hard tissue
	Steam blanching (very short)	Reduced drying time	Extreme risk of fruit disintegration, loss of firmness, juice leakage, flavour change	Moderate capital and operating costs	Analogously to hard tissue
	PEF	Retaining bioactive substances, removing microflora	Juice leakage, possible fruit disintegration	High capital and average operating costs	Analogously to hard tissue
	Carbon dioxide treatment at atmospheric pressure and laser-assisted	Partial enzyme inactivation, colour stabilisation, reducing microflora	Possible flavour changes, uneven effect in heterogeneous materials,	Medium capital and average operating costs	Energy-efficient, eco-friendly, reduction in CO_2_ emission, difficulties with scaling up
	Cold Plasma	Accelerated drying, colour retention	Possible surface oxidation,	Medium-high capital and low operating costs	Low energy consumption, reduced water and solvent use, minimal harmful by-products, eco-friendly and sustainable
Raw materials with a waxy skin (berries, peppers, plums, tomatoes)	Superheated steam blanching	Reduced drying time, reducing oxidation of bioactive substances	Aroma changes with long exposure	Moderate capital and operating costs	Energy-efficient, food waste valorisation
	Carbon dioxide treatment at atmospheric pressure and laser-assisted	Partial enzyme inactivation, colour stabilisation, reducing microflora	Possible flavour changes, uneven effect in heterogeneous materials,	Medium capital and average operating costs	Analogously to soft tissue
	Ultrasound	Reduced drying time, short processing time, retaining bioactive substances	Surface action, possible surface oxidation, aroma changes with long exposure	Moderate capital and operating costs	Analogously to hard tissue
	Enzymatic treatment	Strong disinfection of the surface, no elevated temperature, colour retention	Effect very strongly dependent on technical processing conditions, need for cooling during processing, multiple parameters influencing the effect	Low capital and operating costs	Savings of time, waste valorisation, problem with scaling up
	PEF	Preservation of nutrients, faster drying, reduction in microflora	Microcracks, juice leakage	High capital and average operating costs	Analogously to hard tissue
Small thickness (leafy vegetables, herbs)	Steam blanching	Accelerated drying, preservation of nutrients	Need for individual parameter selection, water phase mediation	Low capital expenditure, low operating costs	Analogously to hard tissue
	UV-C	Shortened drying time, while maintaining nutrients	Uneven heating	High capital expenditures and average operating costs	Sustainable alternative to conventional drying
	Pulse Light	Shortened drying time, microbiological purity	Difficulty in ensuring full surface exposure	Average capital expenditures and average operating costs	Non-thermal and environmentally friendly, energy-efficient, reduction in water use
	Cold plasma	Microbiological purity	Colour changes, risk of overheating	High capital expenditures, high operating costs	Analogously to soft tissue
	Ultrasound	Short processing time, strong antimicrobial activity	Possible surface oxidation,	Average capital expenditures, average operating costs	Analogously to hard tissue
	Carbon dioxide treatment at atmospheric pressure and laser-assisted	Microbiological purity, preservation of bioactive compounds and colour	Aroma changes with long exposure	Average capital expenditures, average operating costs	Analogously to soft tissue
	PEF	Shortened drying time, colour retention, improved oil extraction	Loss of some aromas, need to optimise processing parameters and conditions	High capital expenditures, average operating costs	Analogously to hard tissue

## Data Availability

No new data were created or analysed in this study. Data sharing is not applicable to this article.
